# Degree of Functional Divergence in Duplicates Is Associated with Distinct Roles in Plant Evolution

**DOI:** 10.1093/molbev/msaa302

**Published:** 2020-12-08

**Authors:** Akihiro Ezoe, Kazumasa Shirai, Kousuke Hanada

**Affiliations:** Department of Bioscience and Bioinformatics, Kyushu Institute of Technology, Iizuka, Fukuoka, Japan

**Keywords:** duplicate genes, *Arabidopsis thaliana*, genome evolution, tandem duplication, whole-genome duplication, functional divergence

## Abstract

Gene duplication is a major mechanism to create new genes. After gene duplication, some duplicated genes undergo functionalization, whereas others largely maintain redundant functions. Duplicated genes comprise various degrees of functional diversification in plants. However, the evolutionary fate of high and low diversified duplicates is unclear at genomic scale. To infer high and low diversified duplicates in *Arabidopsis thaliana* genome, we generated a prediction method for predicting whether a pair of duplicate genes was subjected to high or low diversification based on the phenotypes of knock-out mutants. Among 4,017 pairs of recently duplicated *A. thaliana* genes, 1,052 and 600 are high and low diversified duplicate pairs, respectively. The predictions were validated based on the phenotypes of generated knock-down transgenic plants. We determined that the high diversified duplicates resulting from tandem duplications tend to have lineage-specific functions, whereas the low diversified duplicates produced by whole-genome duplications are related to essential signaling pathways. To assess the evolutionary impact of high and low diversified duplicates in closely related species, we compared the retention rates and selection pressures on the orthologs of *A. thaliana* duplicates in two closely related species. Interestingly, high diversified duplicates resulting from tandem duplications tend to be retained in multiple lineages under positive selection. Low diversified duplicates by whole-genome duplications tend to be retained in multiple lineages under purifying selection. Taken together, the functional diversities determined by different duplication mechanisms had distinct effects on plant evolution.

## Introduction

Currently available sequencing technology continues to be used to sequence increasing numbers of complete plant genomes. Analyses of these plant genomes have revealed they contain a higher proportion of duplicates than the genomes of other multicellular organisms (Moore and Purugganan 2005). However, the reason many duplicates are retained in plant genomes remains unclear. Most duplicates tend to be lost immediately after a gene duplication event because mutations accumulate in duplicated genes with redundant functions (Innan and Kondrashov 2010). Some mutations lead to high degree of functional divergence (DFD) of duplicates, and this can be advantageous for adaptive evolution. Therefore, high DFD plays a prominent role in the retention of duplicated genes in plant genomes (Ohno 1970; Kleinjan et al. 2008). Additionally, some ancient duplicated genes appear to have maintained their functions for a long period (Moore and Purugganan 2005; Kafri et al. 2008; Hanada, Kuromori, Myouga, Toyoda, Li, et al. 2009; Kuzmin et al. 2020). Such duplicates functionally compensate for each other and result in a high gene dosage (Gu et al. 2003; Moore and Purugganan 2005). Theoretical studies suggested that the functional compensation by duplicates with redundant functions can contribute to the genetic robustness required to withstand unexpected errors in gene functions and a higher dosage (Wagner 2000; Keane et al. 2014). Furthermore, sequential analyses based on selection pressures inferred by the ratio between the nonsynonymous substitution rate (*K*_A_) and the synonymous substitution rate (*K*_S_) revealed that duplicates with essential functions can be retained as a consequence of natural selection (Hanada, Kuromori, Myouga, Toyoda, Li, et al. 2009; Hanada et al. 2011). Therefore, this redundancy may also be important for the retention of duplicates in plant genomes.

**Fig. 1.**
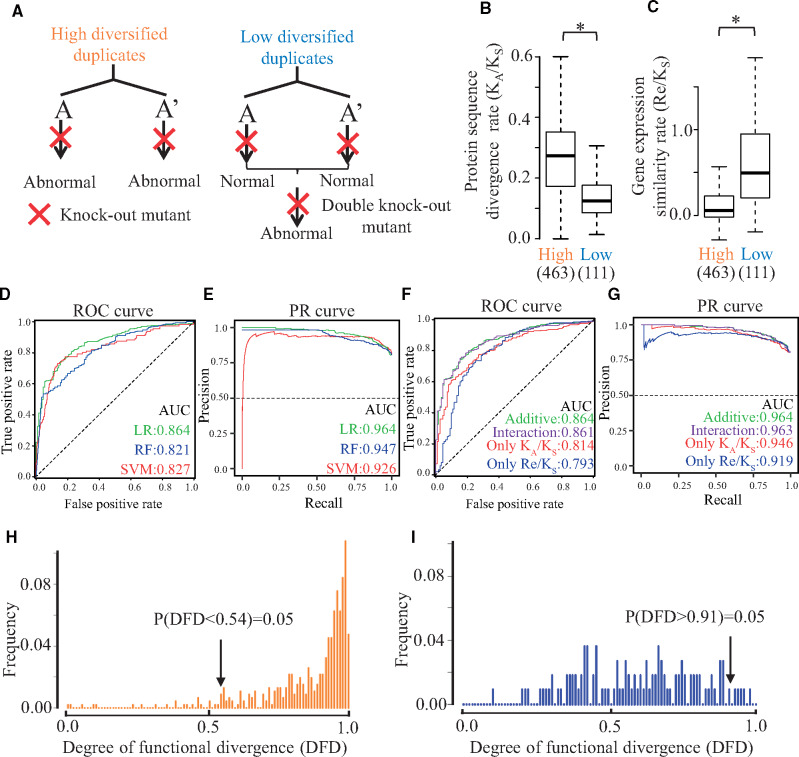
Classification, associated parameters, and prediction of high and low diversified duplicates. (*A*) Single knock-out mutants of each high diversified pair exhibit an abnormal phenotype relative to that of wild-type plant. Double knock-out mutants of two of the low diversified gene pairs exhibit abnormal phenotypes relative to that of the wild-type plant, though the single knock-out mutants of each low diversified gene pair had normal phenotypes. (*B* and *C*) Protein sequence divergence rate and gene expression similarity rate of the duplicate pairs. Protein sequence divergence rates of high and low diversified gene pairs. The protein sequence divergence rate is represented by the ratio of *K*_A_ (number of nonsynonymous substitutions per nonsynonymous site) and *K*_S_ (number of synonymous substitutions per synonymous site). Gene expression similarity rates of the high and low diversified gene pairs. The similarity rate is represented by the ratio of Re (Spearman’s correlation coefficient for the expression levels among 634 conditions) to *K*_S_ for the duplicate pairs. The distributions of the *K*_A_/*K*_S_ and Re/*K*_S_ ratios are presented in box plots with the solid horizontal line indicating the median value, the box representing the interquartile range (25–75%), and the dotted line indicating the first to the 99th percentile. The asterisk indicates a significant difference (*P *<* *0.05, two-tailed Wilcoxon rank sum test). Numbers in parenthesis represent the number of duplicate pairs. (*D*) Receiver operating characteristic (ROC) curves in our prediction models. Three colored lines represent receiver operating characteristic (ROC) curves of LR, Support Vector Machine (SVM), and Random Forest (RF) algorithms. The area under the curve (AUC) values were calculated by the best prediction model in each algorithm. (*E*) Precision–Recall (PR) Curve in our prediction models. The area under the curve (AUC) values were calculated by the best prediction model in each algorithm. (*F*) ROC curves in our prediction models. ROC curves were generated in four types of formulas based on LR algorithms. (*G*) Precision–Recall (PR) Curve in our prediction models. (*H*) Histogram of the inferred degree of functional divergence (DFD) in high diversified duplicates of the training data. The inferred DFD was calculated for 493 high diversified gene pairs. The inferred DFD was mostly very high. The bottom 5% of the inferred DFD values were <0.54 (i.e., low DFD at 5% false-positive rate). (*I*) Histogram of the inferred DFD for the low diversified duplicates of the training data. The inferred DFD was calculated for 111 low diversified gene pairs. The top 5% of DFD values were >0.91 (i.e., high DFD at 5% false-positive rate).

Some retained duplicates exhibit functional divergence, whereas others maintain redundant functions with the paralogs. The functional divergence of duplicates can be inferred based on the phenotypes of knock-out mutants in *Arabidopsis thaliana* (fig. 1*A*) (Hanada, Kuromori, Myouga, Toyoda, and Shinozaki 2009; Hanada, Kuromori, Myouga, Toyoda, Li, et al. 2009; Chen et al. 2010). When single knock-out mutants of individual duplicates exhibit abnormal phenotypes under a specific condition, the duplicates are not compensated by the other gene copies. In our analysis of abnormal phenotypes, we included only the morphological changes relative to the wild-type controls under optimal growth conditions for *A. thaliana*. The associated knocked out genes are likely to play significant roles in plant development. Consequently, these knock-out mutants are likely to exhibit abnormal phenotypes under any conditions. Such duplicates with functional divergence are herein defined as high diversified duplicates. When a double knock-out mutant of duplicates exhibits an abnormal phenotype in cases where an abnormal phenotype was not observed for single knock-out mutants of each duplicate, the duplicates share redundant functions with the other gene copy. We did not define such duplicates as redundant duplicates because single knock-out mutants may exhibit abnormal phenotypes under different conditions not assayed in corresponding experiments. An earlier study involving yeast revealed that most duplicates induce abnormal phenotypes under multiple conditions (Hillenmeyer et al. 2008). Thus, the redundant duplicates identified under optimal conditions may exhibit some characteristics of diversified duplicates under other conditions. These duplicates are herein defined as low diversified duplicates. The effects of gene dosage due to gene duplications should also be considered. Even if both duplicates have essentially the same function, knocking out one of the duplicates may induce abnormal phenotypic changes because of a decrease in gene dosage (Blanc and Wolfe 2004). In the present study, such duplicates are categorized as high diversified duplicates. Thus, the classification based on morphological phenotypes is not ideal if morphological phenotypes are highly influenced by gene dosage effects.

We previously determined that high or low diversified duplicates can be inferred by the protein sequence divergence rate or the gene expression similarity rate (Hanada, Kuromori, Myouga, Toyoda, and Shinozaki 2009; Hanada, Kuromori, Myouga, Toyoda, Li, et al. 2009). However, it is difficult to examine the DFD in duplicates by these two factors manually. In another study, researchers attempted to generate a prediction model to classify high and low diversified duplicates which can quantitatively examine the DFD (Chen et al. 2010). However, the model essentially randomly classified high and low diversified duplicates because it did not consider the protein sequence divergence rate or the gene expression similarity rate when assessing functional divergence.

In the present study, after carefully updating the classification (i.e., high or low diversified duplicates), we generated 463 high and 111 low diversified duplicate pairs in *A. thaliana* as a training data. Using the training data, we developed a model for predicting high or low diversified duplicates in a pair of duplicates based on the rate of substitutions in protein sequences and the divergence rate of expression patterns. After defining a classification threshold (high or low diversified duplicates), we used the model for inferring the high or low diversified duplicate pairs of the closest *A. thaliana* paralogs. Focusing on four duplicate pairs classified as high or low diversified duplicates by our model, we validated the classification based on the phenotypic effects of gene knock-downs in transgenic plants. Finally, to elucidate the roles of high or low diversified duplicates, we extensively examined the functional bias between high and low diversified duplicates in *A. thaliana*, and examined the persistence of high and low diversified duplicates during evolution.

## Results and Discussion

### Classification of Duplicated Genes as High or Low Diversified Duplicates

Our previous studies involved 492 duplicate pairs that were classified as high or low diversified pairs based on phenotypes of knock-out mutants under only optimal growth condition of *A. thaliana* (fig. 1*A*) (Hanada, Kuromori, Myouga, Toyoda, and Shinozaki 2009; Hanada, Kuromori, Myouga, Toyoda, Li, et al. 2009). We additionally identified 1,149 duplicate pairs with morphological phenotypic data from knock-out plants for a total of 1,641 duplicate pairs on the same condition. Our collected genes are likely to play significant roles in plant developments. Of these 1,641 pairs, we removed 1,067 unreliable duplicate pairs (identity < 30%, coverage < 50%, and *K*_S_ > 3). The remaining 574 duplicate pairs were classified as 463 high and 111 low diversified duplicate pairs, respectively (supplementary table S1, Supplementary Material online). Phenotypic effects were classified into several groups (e.g., seedling, vegetative, reproductive, lethal, and gametophyte) based on previously described categories (Hanada, Kuromori, Myouga, Toyoda, and Shinozaki 2009; Lloyd and Meinke 2012). A comparison between the single and double knock-out mutants indicated the phenotypic categories were similar (supplementary fig. S1*A* and *B*, Supplementary Material online).

Differences in protein sequences and gene expression have been proposed as one of the main determinants of functional divergence (Van de Peer et al. 2001; Jordan et al. 2004). We previously examined that the divergence rate of protein sequences encoded by duplicate gene pairs were significantly higher for the high diversified duplicates than for the low diversified duplicates (Hanada, Kuromori, Myouga, Toyoda, and Shinozaki 2009). The protein sequence divergence rates can be inferred from the selection pressure on the protein-coding sequences, which is represented by *K*_A_/*K*_S_ (supplementary document 1, Supplementary Material online). High and low *K*_A_/*K*_S_ ratios indicate high and low protein divergence rates at the same duplication age, respectively. Regarding our newly generated 574 duplicate pairs, we revealed the same trend in which *K*_A_/*K*_S_ ratios were significantly higher for the high diversified pairs than for the low diversified pairs (fig. 1*B*;*P = *7.21 × 10^*−*^^25^, two-tailed Wilcoxon rank sum test). The difference in gene expression can be evaluated by accumulated transcriptome data. Here, the similarity in the expression of a duplicate gene pair was determined based on Spearman’s correlation coefficient for the gene expression levels of a microarray analysis of 634 conditions collected from The Arabidopsis Information Resource (TAIR) database (version 10, https://www.arabidopsis.org, last accessed Octobor 16, 2020) (Re; supplementary document 1, Supplementary Material online). To examine the expression similarities at the same duplication ages, Re was divided by *K*_S_. In our 574 pairs, the gene expression similarity rate (Re/*K*_S_) was significantly higher for the low diversified pairs than for the high diversified pairs (fig. 1*C*;*P = *7.56 × 10^−22^, two-tailed Wilcoxon rank sum test), which was consistent with the results of a previous study (Hanada, Kuromori, Myouga, Toyoda, and Shinozaki 2009). We also tried to use other divergence rates associated with functional divergence as explanatory variables. However, they did not differ significantly between the high and low diversified pairs (supplementary document 1 and fig. S2*A–C*, Supplementary Material online). Thus, our newly generated 574 duplicate pairs were effectively classified as high and low diversified duplicates based on *K*_A_/*K*_S_ and Re/*K*_S_.

### Generalized Linear Model to Classify Duplicates as High or Low Diversified Duplicates

In the current study, we used these 574 duplicate pairs as training data for predicting the functionalization or redundancy of duplicated genes. To accurately classify 574 duplicate pairs as high or low diversified duplicates, we developed three prediction models based on logistic regression (LR), random forest (RF), and support vector machine (SVM) algorithms with two explanatory variables (*K*_A_/*K*_S_: protein sequence divergence rate, Re/*K*_S_: gene expression similarity rate). For the three algorithms, the objective variables 0 and 1 corresponded to high and low diversified duplicates, respectively. The best prediction models for each algorithm were chosen. The three models were compared by calculating the area under the curve-receiver operating characteristic (AUC-ROC) value (fig. 1*D*), and the area under precision recall curve (AU-PRC) value (fig. 1*E*). The AUC-ROC values were 0.864 (LR), 0.821 (RF), and 0.827 (SVM). The AU-PRC values were 0.964 (LR), 0.947 (RF), and 0.926 (SVM). These results indicate that the LR algorithm was the most appropriate for predicting high or low diversified duplicates. The model generated with the LR algorithm was used in the following analyses.

The best AUC-ROC value was 0.86, which is much higher than the corresponding value (0.56) for the model developed by Chen et al. (2010). There are two possible reasons why our model produced a higher AUC-ROC value. First, the training data used by Chen et al. (2010) were unreliable because 35% (23/65) of the classifications did not correspond to the reported classification (supplementary table S1, Supplementary Material online). Second, the model developed by Chen et al. (2010) lacks crucial variables, namely the protein sequence divergence rate and the gene expression similarity rate. Additionally, the other previous reports tend to examine the functional divergence of duplicates by only protein sequence divergence (*K*_A_/*K*_S_ ratio) (Shirai et al. 2014; Hanada et al. 2018). We also evaluated whether a model based on both *K*_A_/*K*_S_ and Re/*K*_S_ is better than models based on either *K*_A_/*K*_S_ or Re/*K*_S_. We compared the AUC-ROC and AU-PRC values between the model based on *K*_A_/*K*_S_ and Re/*K*_S_ and the models based on only one parameter (*K*_A_/*K*_S_ or Re/*K*_S_) (fig. 1*F* and *G*). Both AUC-ROC and AU-PRC values were the highest in a model applying both *K*_A_/*K*_S_ and Re/*K*_S_, implying that a model based on both *K*_A_/*K*_S_ and Re/*K*_S_ is better than models based on either *K*_A_/*K*_S_ or Re/*K*_S_.

The DFD can be inferred from the best formula by LR analysis. As DFD is close to 1, the functional divergence is enlarged. As DFD is close to 0, the functional divergence is minimized. To minimize the erroneous assigning of high or low diversification to anonymous duplicate pairs, we used a 5% false-positive rate as a threshold. The DFD thresholds were 0.91 and 0.54 for the duplicates assigned as high and low diversified, respectively (fig. 1*H* and *I*). Because a low false-positive rate is associated with a high false-negative rate, our classifications tend to have a high false-negative rate (i.e., 42% and 61% for assigning high and low diversification, respectively).

### Classification of Recently Duplicated Genes as High or Low Diversification

We applied our model to classify anonymous duplicate pairs as high or low diversified. To examine the functional divergence of *A. thaliana* duplicate pairs, we focused on 4,017 recently duplicated pairs in *A. thaliana* (supplementary document 2, Supplementary Material online). Of these pairs, we classified 1,052 (26.1%) as high and 600 (14.9%) as low diversified pairs. However, 2,465 (59%) of the duplicate pairs were not classified as either high or low diversified because of goal of minimizing incorrect classifications.

It should be noted that knocking out one of the duplicates might induce abnormal phenotypic changes by gene dosage (Blanc and Wolfe 2004). Such duplicates should be designated as low diversified duplicates but the duplicates might be predicted as high diversified duplicates in our model. If the errors were observed in our prediction, truly low diversified genes tend to be frequently predicted as high diversified duplicates. To examine whether the trend is observed or not, we focused on five high and 22 low diversified duplicates assigned in training data but the 27 duplicates were incorrectly predicted as opposite diversified duplicates in our model. The proportion of the incorrectly predicted duplicates was not significantly different between high and low diversified duplicates (5/111 = 4.50% vs. 22/463 = 4.75%, *P *=* *0.916, χ^2^ test), indicating that gene dosage effects are negligible in our prediction.

### Experimental Validation Based on Transgenic Plants

To validate our classifications, we focused on two duplicate pairs assigned as high diversified and two duplicate pairs assigned as low diversified (supplementary document 3 and fig. S3, Supplementary Material online). For all eight genes, we generated transgenic plants in which a single gene was knocked down. We expected that the phenotypes of the resulting transgenic plants for the genes assigned as high and low diversified pairs would be abnormal and normal relative to the wild-type, respectively. We also generated two transgenic plants in which two paralogs assigned as low diversified duplicates were knocked down. These transgenic plants were expected to be phenotypically abnormal relative to the wild-type. To determine the functional roles of these genes clearly, it was desirable to examine phenotypic effects in various conditions or developmental stages. In the present analyses, we focused on phenotypic changes under only seedling stage because seedling developments are highly sensitive to genetic changes rather than any other organs (Mayer et al. 1991). The transgenic seedlings were compared regarding the root length, hypocotyl length, and the root length:hypocotyl length ratio because root and hypocotyl morphological traits are considered to be representative phenotypic characteristics of seedlings (Lachowiec et al. 2015). All the target genes were highly expressed in seedling stages (supplementary table S2, Supplementary Material online). The effects of knocking down target genes in transgenic plants were confirmed by qRT-PCR (supplementary document 4 and table S3, Supplementary Material online). The expression of all target genes was significantly suppressed in the transgenic plants (supplementary fig. S4*A* and table S4, Supplementary Material online); however, there were no changes to the expression of the nontarget genes (supplementary fig. S4*B* and table S4, Supplementary Material online). To examine phenotypic differences, we performed Kolmogorov–Smirnov test to examine whether hypocotyl length, root length, or root/hypocotyl ratio follows normal distribution or not. As a result, all *P* values were above 0.05 (supplementary table S5, Supplementary Material online), indicating that each of phenotypic effects follows normal distribution. Therefore, we used *t*-test to examine phenotypic differences in the following analyses.

**Fig. 2.**
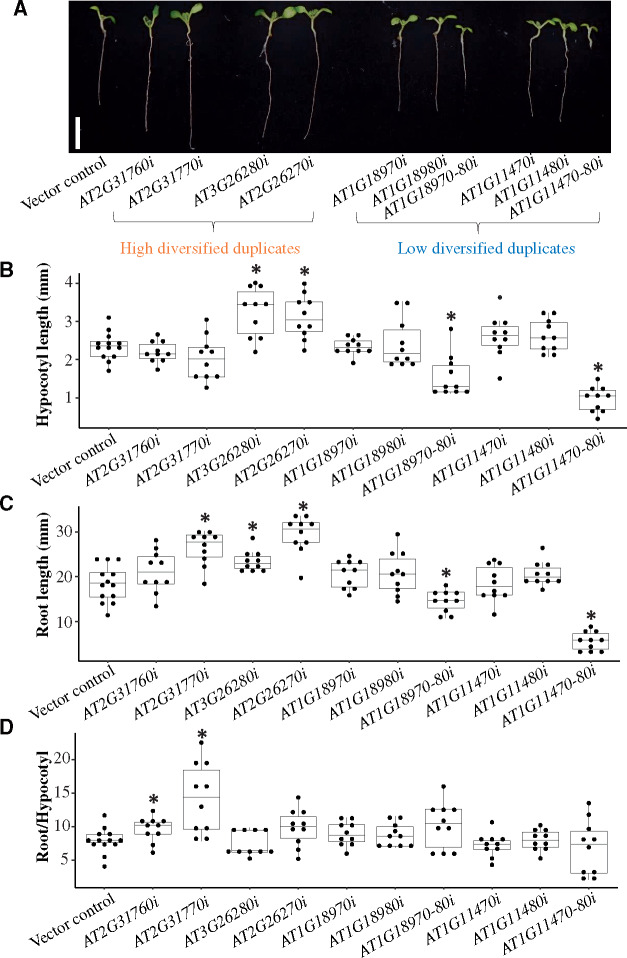
Effects of the knock-down of high and low diversified duplicates. (*A*) The white bar represents 1 cm. Transgenic plants overexpressing only the *BAR* gene were used as the vector control. Transgenic plants with a single gene knocked down (AT2G31760, AT2G31770, AT3G26270, AT3G26280, AT1G18970, AT1G18980, AT4G11470, and AT4G11480) are indicated by AT2G31760i, AT2G31770i, AT3G26270i, AT3G26280i, AT1G18970i, AT1G18980i, AT4G11470i, and AT4G11480i, respectively. Additionally, AT2G31760, AT2G31770, AT3G26270, and AT3G26280 are high diversified duplicates, whereas AT1G18970, AT1G18980, AT4G11470, and AT4G11480 are low diversified duplicates. Transgenic plants with two of the low diversified gene pairs knocked down (AT1G18970 and AT1G18980 as well as AT4G11470 and AT4G11480) are indicated by AT1G18970-80i and AT4G11470-80i, respectively. (*B*) Hypocotyl length, (*C*) root length, and (*D*) root length:hypocotyl length ratio for the 11 transgenic plants presented in panel (*A*). Phenotypic differences were determined based on ten biological replicates. The distributions of phenotypic differences are presented in box plots with the solid horizontal line indicating the median value, the box representing the interquartile range (25–75%), and the dotted line indicating the first to the 99th percentile. The asterisk indicates a significant difference compared with the vector control (*P *<* *0.05, two-tailed Student’s *t*-test).

Four transgenic plants were generated for two high diversified pairs (fig. 2*A*; AT2G31760 and AT2G31770; AT3G26270 and AT3G26280). There were no significant differences in the hypocotyl length between the transgenic plants for AT2G31760 and AT2G31770 and the control plants (fig. 2*B*, *P = *0.29 for AT2G31760 and *P *=* *0.15 for AT2G31770, two-tailed Student’s *t*-test). The transgenic plants for AT2G31770 had longer roots than the control plants (fig. 2*C*;*P = *1.02 × 10^−4^, two-tailed Student’s *t*-test), whereas the root lengths of the transgenic plants for AT2G31760 were not significantly different from the control plant (fig. 2*C*;*P = *0.15, two-tailed Student’s *t*-test). The root length:hypocotyl length ratio was significantly greater for both transgenic plants (fig. 2*D*;*P = *4.21 × 10^−2^ for AT2G31760 and *P = *4.01 × 10^−3^ for AT2G31770, two-tailed Student’s *t*-test). Additionally, the transgenic plants for AT3G26280 and AT3G26270 had longer hypocotyls (fig. 2*B*;*P = *1.48 × 10^−3^ for AT3G26280 and 2.05 × 10^−3^ for AT3G26270, two-tailed two sample Student’s *t*-test) and longer roots (fig. 2*C*;*P = *1.41 × 10^−3^ for AT3G26280 and 5.10 × 10^−6^ for AT3G26270, two-tailed Student’s *t*-test) than the control plants, but there were no significant differences in the root length:hypocotyl length ratios of the transgenic and the control plants (fig. 2*D*;*P = *0.58 for AT3G26280 and 0.08 for AT3G26270, two-tailed Student’s *t*-test). These results indicated that, as expected, the knock-down of the four high diversified duplicates induced abnormal phenotypic changes.

We analyzed four transgenic plants for two low diversified pairs (fig. 2*A*; AT1G18970 and AT1G18980; AT4G11470 and AT4G11480). There were no differences in the hypocotyl and root lengths and the root length:hypocotyl length ratios between the control plants and the transgenic plants for AT1G18970, AT1G18980, AT4G11470, and AT4G11480 (fig. 2*B–D*; *P *=* *0.97, 0.14, and 0.22 for AT1G18970; 0.72, 0.22, and 0.29 for AT1G18980; 0.21, 0.95, and 0.38 for AT4G11470; and 0.11, 0.13, and 0.92 for AT4G11480, two-tailed Student’s *t*-test). Four transgenic plants, there were no differences in the variances of the hypocotyl length, root length, or root length:hypocotyl length ratio between the control and transgenic plants (supplementary table S5, Supplementary Material online, *P *>* *0.05, two-tailed *F*-test).

However, the two transgenic plants in which the duplicate pairs were knocked down had shorter hypocotyls (fig. 2*B*;*P = *1.52 × 10^−3^ for AT1G18970 and AT1G18980 and 6.09 × 10^−9^ for AT4G11470 and AT4G11480, two-tailed Student’s *t*-test) and shorter roots (fig. 2*C*;*P = *1.29 × 10^−3^ for AT1G18970 and AT1G18980 and 1.19 × 10^−8^ for AT4G11470 and AT4G11480, two-tailed Student’s *t*-test) than the control plants. These results indicated that knocking down a single low diversified duplicate had no significant effect on the phenotype of the transgenic plants. In contrast, knocking down both low diversified duplicates resulted in transgenic plants with an abnormal phenotype. The same phenomena were observed in other independent lines (supplementary fig. S5, Supplementary Material online). Thus, the classifications of duplicated genes based on our prediction model were experimentally validated.

### Computational Validation Based on Gene Annotations

To further validate our prediction model based on gene functions, we examined the gene ontology (GO) terms assigned to the high and low diversified duplicates (supplementary document 5, Supplementary Material online). We expected that diverse GO terms would tend to be assigned to the high diversified pairs, but not the low diversified pairs. An analysis of the proportion of shared GO terms for the high and low diversified pairs revealed that the proportion was lower for the high diversified pairs (supplementary fig. S6*A*, Supplementary Material online; *P = *2.74 × 10^−33^, two-tailed Wilcoxon rank sum test). We also expected that functional domains (FD) tended to be shared in the low diversified pairs, but not in the high diversified pairs. The proportion of shared FDs tended to be lower in the high diversified pairs than that in the low diversified pairs (supplementary fig. S6*B*, Supplementary Material online; *P *=* *4.26 × 10^−2^, two-tailed Wilcoxon rank sum test). These results provide further evidence for the accuracy of our prediction model.

However, high and low proportions of shared GO terms are expected for recent and older duplicates, respectively. If the duplication timing leads to differences between high and low diversified pairs, the high diversified pairs would tend to be duplicated more recently than the low diversified pairs. Thus, we compared the *K*_S_ values of the high and low diversified pairs. The lack of a significant difference (supplementary fig. S6*C*, Supplementary Material online; *P *=* *0.33, two-tailed Wilcoxon rank sum test) indicated that the high and low diversified pairs do not have any strong bias in duplication timing. Additionally, the GO terms assigned to a large gene family are counted multiple times. To minimize the effects of large gene families, we only analyzed recently duplicated pairs. Thus, there was no strong bias in the duplication timing and gene family size when comparing the shared GO terms between the high and low diversified pairs.

To validate whether our approach is applicable or not, we focused phenome analyses in 338 duplication pairs (Jover-Gil et al. 2014). Out of 338 pairs, 40 pairs were used as recently duplicate pairs in the present study. Our prediction model inferred two high and five low diversified pairs among 40 pairs. The phenome analyses identified six pairs as the same diversified categories but one pair as the opposite categories. Therefore, the accuracy of our prediction model was estimated to be 86% (6/7). Thus, our approach seems to be applicable in the other reports.

### Functional Bias between the High and Low Diversified Duplicates

To assess the functional bias between the high and low diversified duplicates, we examined the corresponding enriched GO terms. Out of 648 and 579 GO terms assigned to high and low diversified duplicates, 71 and 239 GO terms were significantly enriched, respectively. The enriched GO terms among the high diversified duplicates included cytidine metabolic process (GO: 0046087), defense response (GO: 0006952), and lipid transport (GO: 0006869) (supplementary table S6, Supplementary Material online). The cytidine metabolic process and lipid transport are reportedly important for plant defense responses (Brightbill 1999). Thus, it is likely that high diversified duplicates may have increased the diversity of plant defense responses during evolution. The over-representation of immune responses among the high diversified duplicates may be because genes associated with immune responses are likely to have novel functions to provide protection against various pathogens.

**Fig. 3.**
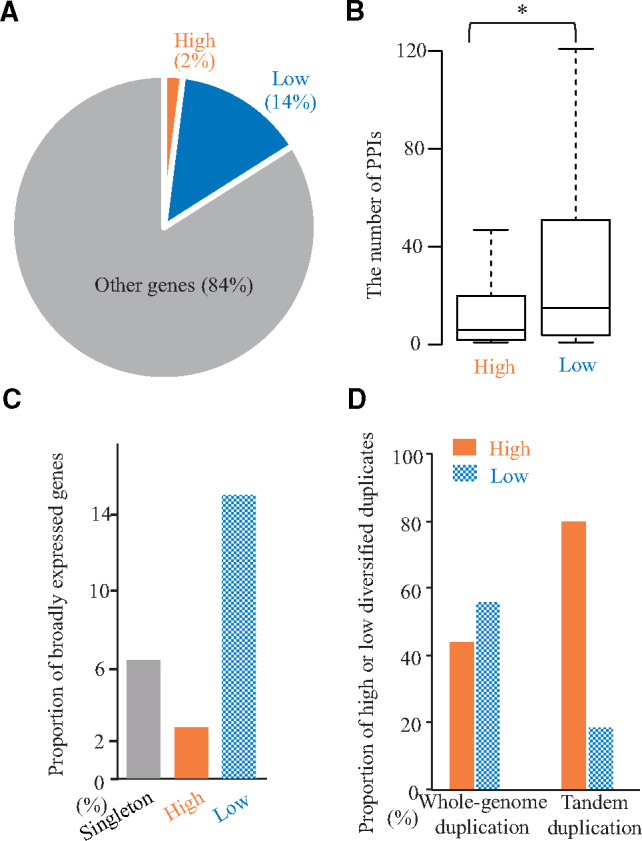
Differences in the roles of duplicates with high and low diversified duplicates. (*A*) The core genes were defined as highly conserved genes in eukaryotic genomes. The pie chart area indicates the proportion of high and low diversified duplicates of duplicates as well as the other core genes. (*B*) The distributions of the number of protein–protein interactions (PPIs) are presented in box plots with the solid horizontal line indicating the median value, the box representing the interquartile range (25–75%), and the dotted line indicating the first to the 99th percentile. The asterisk indicates a significant difference (*P *<* *0.05, two-tailed Wilcoxon rank sum test). (*C*) The proportions of broadly expressed genes among summation of broadly and specifically expressed genes for the high and low diversified duplicates and singletons are presented as bar plots. (*D*) The proportions of whole-genome and tandem duplication events for the high and low diversified duplicates are presented as bar plots.

The enriched GO terms among the low diversified duplicates were largely classified into three categories such as biogenesis/catabolism in molecular complex, cellular component biogenesis, and metabolism of primary metabolites (supplementary table S6, Supplementary Material online). Biogenesis/catabolism in molecular complex includes ribosome biogenesis (GO: 0042254), Translation (GO: 0006412), RNA modification (GO: 0009451), RNA metabolic process (GO: 0016070), Protein metabolic process (GO: 0019538), Ubiquitin-dependent protein catabolic process (GO: 0006511), Modification-dependent protein catabolic process (GO: 0019941), and Modification-dependent macromolecule catabolic process (GO: 0043632). Cellular component biogenesis includes Cell morphogenesis (GO: 0000902), Cellular component assembly (GO: 0022607), Cellular component biogenesis (GO: 0022607), Cellular component morphogenesis (GO: 0032989), Cellular component organization (GO: 0016043), Cellular component organization or biogenesis (GO: 0071840), Cellular macromolecular complex assembly (GO: 0034622), Cellular macromolecule metabolic process (GO: 0044260), and Cellular protein complex assembly (GO: 0043623). Metabolism of primary metabolites includes Organic substance metabolic process (GO: 0071704), Macromolecule metabolic process (GO: 0043170), Primary metabolic process (GO: 0044238), Carbohydrate metabolic process (GO: 0005975), Hexose metabolic process (GO: 0019318), and Glucose metabolic process (GO: 0006006). These functions may be essential in most living organisms. To clarify the importance of high and low diversified duplicates, we examined core genes that are highly conserved in eukaryotic genomes (Parra et al. 2007, supplementary document 6, Supplementary Material online). Specifically, the core genes included higher proportion of low diversified duplicates (14%) than that of genes with high diversified duplicates (2%; fig. 3*A*, P* = *1.42 × 10^−16^, χ^2^ test), implying that essential genes tend to be low diversified duplicates. We also examined the relationship between the low diversified duplicates and the protein–protein interaction (PPI) data (supplementary document 6, Supplementary Material online), which indicates the relevance of essential signal transductions (He and Zhang 2005). The number of PPIs associated with low diversified duplicates tended to be greater than the number of PPIs related to high diversified duplicates (fig. 3*B*;*P = *2.12 × 10^−11^, two-tailed Wilcoxon rank sum test). These results indicate that the duplicates associated with essential signal transductions tend to be low diversified duplicates.

It is likely that genes involved in essential signaling pathways are expressed under broad conditions, whereas genes that underwent a recent functionalization, such as those involved in biotic stress responses, tend to be expressed under specific conditions (Hanada et al. 2008; Jiang et al. 2013). To examine the distribution of expression profiles, we compiled 2,001 specifically and 115 broadly expressed genes based on the available microarray data for 634 conditions (supplementary document 6, Supplementary Material online). The ratio between high and low diversified duplicates (180/174) in specifically expressed genes was significantly higher than the ratio in broadly expressed genes (fig. 3*C*; 5/31, *P *=* *5.00 × 10^−5^, χ^2^ test) indicating that high and low diversified duplicates tended to be specifically and broadly expressed, respectively. These results are consistent with our expectations.

The low diversified duplicates were associated with essential signaling pathways. The inhibition of essential signaling pathways can be lethal for plants. Therefore, preventing the deletion of these pathways during evolution might be advantageous for plants. Indeed, previous studies confirmed that functional redundancy can help to mitigate the effects of frequent deleterious mutations (Tanaka et al. 2009; Kuzmin et al. 2020). Another study showed correlation between sequence divergence from closest paralog and essentiality of duplicated genes (Makino et al. 2009). Theoretical studies revealed that the functional compensation by duplicated genes during evolution may be advantageous when there are frequent unexpected errors in gene functions (Nowak et al. 1997). Animal has primordial germ cells for propagation in early developmental stages, but plant does not have such strictly diversified cells in early developmental stages. Instead, plant germ cells can arise from somatic cells, including those affected by somatic mutations (Schoen and Schultz 2019). In other words, unexpected errors in gene functions tend to occur more frequently in plant germ cells than in animal germ cells. Thus, low diversified duplicates may be crucial for improving the functional compensation at a genome-wide scale.

We found new candidates of five explanatory variables (the number of shared GOs, the number of shared FDs, PPI, core genes, and broadly expressed genes) to classify high and low diversified duplicates. However, out of five variables, two variables (core genes and broadly expressed genes) are not assigned in most duplicates. Therefore, we focused on three variables such as the number of shared GO terms, the number of shared FD, and the number of PPI which are assigned in most duplicates. To examine the explanatory weights of these variables, we calculated relative importance of each variable under LR algorithm (Team R Development Core 2013). To classify duplicates into high and low diversified duplicates, the most important variable and the second one were *K*_A_/*K*_S_ and Re/*K*_S_, respectively (supplementary fig. S7*A*, Supplementary Material online). The third, fourth, and fifth were FD, GO, and PPI, respectively (supplementary fig. S7*A*, Supplementary Material online). Relative importance of *K*_A_/*K*_S_ and Re/*K*_S_ was extraordinary higher than that of either FD, GO, or PPI. We then generated five models based on LR algorithm with each of five variables. AUC-ROC values were calculated in models based on *K*_A_/*K*_S_, Re/*K*_S_, FD, GO, and PPI were 0.814, 0.793, 0.600, 0.571, and 0.567, respectively (supplementary fig. S7*B*, Supplementary Material online). AU-PRC values are 0.946, 0.919, 0.861, 0.836, and 0.830 in *K*_A_/*K*_S_, Re/*K*_S_, FD, GO, and PPI, respectively (supplementary fig. S7*C*, Supplementary Material online). These results indicate that two variables (*K*_A_/*K*_S_ and Re/*K*_S_) are useful parameters to infer functional divergence of duplicates, but it is unlikely that the other variables (FD, GO, and PPI) contribute to the classification of functional divergence in duplicates. Nevertheless, we generated a model based on five variables (*K*_A_/*K*_S_, Re/*K*_S_, FD, GO, and PPI), and compared AUC-ROC and AU-PRC values based on five variables with those based on only *K*_A_/*K*_S_ and Re/*K*_S_. Consequently, AU-PRC value (0.96367) based on five variables is slightly higher than that (0.96360) based on *K*_A_/*K*_S_ and Re/*K*_S_ but AUC-ROC value (0.86333) based on five variables is slightly lower than that (0.86446) based on *K*_A_/*K*_S_ and Re/*K*_S_ (supplementary fig. S7*B* and *C*, Supplementary Material online). From these results, we concluded that FD, GO, and PPI do not contribute to the classification of functional divergence effectively. Therefore, we used the model based on *K*_A_/*K*_S_ and Re/*K*_S_ in the following analyses.

### Duplication Mechanisms Associated with High or Low Diversified Duplicates

Most duplicates are derived from tandem duplication or whole-genome duplication events (Panchy et al. 2016). Tandem duplications tend to be associated with lineage-specific functions like responses to biotic stresses or environmental stimuli (Hanada et al. 2008). Such lineage-specific functions are likely to undergo functionalization. Additionally, to avoid serious errors, regulatory genes, including those encoding transcription factors, tend to be retained after a whole-genome duplication event, as proposed in the gene balance hypothesis (Van de Peer et al. 2017). It is likely that the genes retained after a whole-genome duplication contribute to the functional compensation via the maintenance of redundant functions. Thus, the duplication mechanisms may induce the differences related to high and low diversified duplicates. On the basis of the assignment of tandem duplications or whole-genome duplications (DeLuna et al. 2008; Shirai et al. 2017), we compared the ratio of tandem duplication to whole-genome duplication in high and low diversified duplicates. The ratio (tandem/whole-genome duplication) of high diversified duplicates (705/347 = 2.0) was significantly higher than that of the low diversified duplicates (supplementary document 6, Supplementary Material online, 160/440 = 0.36, fig. 3*D*;*P *=* *3.07 × 10^−30^, χ^2^ test). These results indicate that duplicates derived from tandem duplications tend to undergo high diversified functionalization, whereas the duplicates resulting from whole-genome duplications tend to undergo low diversified functionalization. Therefore, the DFD is affected by the mechanisms underlying the duplication event.

### Retention Bias of the Orthologs of *A. thaliana* Duplicates with High or Low Diversified Duplicates

It is likely that duplicate genes retained in multiple lineages largely contribute to adaptive evolution (Wuchty et al. 2003). To assess the retention bias, we identified the duplication events occurring before the speciation from the common ancestor of Brassicaceae lineage and examined the existence of orthologous genes in closely related species (supplementary document 7, Supplementary Material online). Phylogenetic analyses of 1,047 duplicate pairs in 32 plant species (supplementary fig. S8*A* and table S7, Supplementary Material online) revealed 991 duplicate pairs that were generated before the speciation from the common ancestor of Brassicaceae lineage (supplementary fig. S8*B*, Supplementary Material online). Among these 991 duplicate pairs, 980 resulted from duplication events that occurred after the speciation of the Malvidae lineage. Additionally, the 991 duplicate pairs were classified as 505 high and 486 low diversified duplicate pairs.

**Fig. 4.**
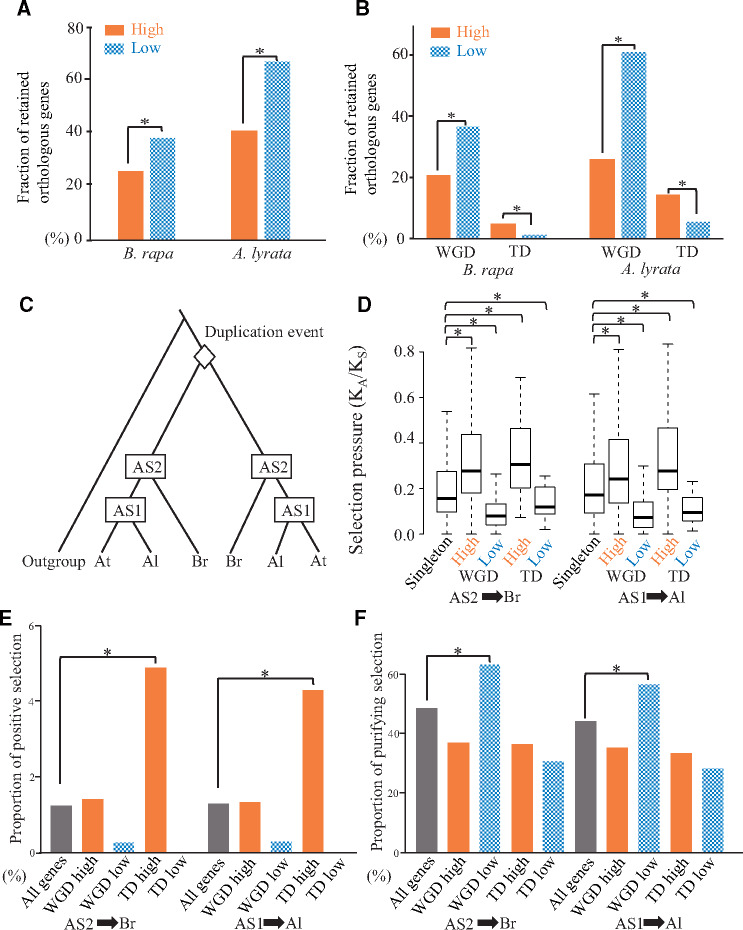
Comparison of the retention rates and selective pressures of the orthologous genes in *Brassica rapa* and *Arabidopsis lyrata*. (*A* and *B*) The bars filled and with checkered pattern represent the retention rates of genes assigned to be high and low diversified duplicates, respectively. WGD and TD represent whole-genome and tandem duplicates, respectively. (*C*) Topology of a phylogenetic tree comprising 991 pairs of *A. thaliana* duplicates whose duplication events occurred before the divergence between the Brassicaceae and Arabidopsis lineages. The rhombus represents the duplication events for a pair of *A. thaliana* duplicates. Additionally, AS1, AS2, At, Al, and Br represent the ancestral node between *A. thaliana* and *A. lyrata* as well as the ancestral node among *A. thaliana*, *A. lyrata*, and *B. rapa*, respectively. (*D*) Selection pressures in *B. rapa* and *A. lyrata* lineages. The *K*_A_ and *K*_S_ represent the nonsynonymous substitution rate and synonymous substitution rate, respectively, in two branches (AS1 to Br and AS2 to Al). The distributions of the *K*_A_/*K*_S_ values are presented in box plots with the solid horizontal line indicating the median value, the box representing the interquartile range (25–75%), and the dotted line indicating the first to the 99th percentile. The asterisk represents a significant difference (*P* < 0.05, two-tailed Wilcoxon rank sum test). (*E* and *F*) Selection pressures in *B. rapa* and *A. lyrata* lineages. The bars filled and with checkered pattern represent the proportion of branches at which positive or purifying selections were detected. High and low represent high and low diversified duplicates, respectively. WGD and TD represent whole-genome and tandem duplicates, respectively.

Assuming that orthologous genes maintain the same function among three species, the retention of high and low diversified duplicates can be tested in the following analysis. We searched for orthologous pairs in either *Brassica rapa* or *Arabidopsis lyrata* (supplementary document 8, Supplementary Material online). The retention rate was significantly higher for the low diversified duplicates than for the high diversified duplicates of orthologous pairs in *B. rapa* (fig. 4*A* and supplementary table S8, Supplementary Material online; 38% vs. 26%, *P = *4.58 × 10^−7^, χ^2^ test) as well as in *A. lyrata* (fig. 4*A* and supplementary table S8, Supplementary Material online; 67% vs. 41%, *P = *3.82 × 10^−24^, χ^2^ test). These results indicate that low diversified duplicates tend to be retained in different lineages more than the high diversified duplicates as a whole. Thus, it is likely that functional compensation via low functionalized duplicates play essential roles in Brassicaceae lineage at genomic scale.

Duplicates derived from tandem and whole-genome duplications exhibit lineage-specific expansion and convergent retention in different lineages, respectively (Panchy et al. 2016). It is expected that whole-genome duplicates tend to be retained more frequently in multiple lineages than tandem duplicates. Indeed, the retention rates were higher for the whole-genome duplicates than for tandem duplicates (64% vs. 31% in *B. rapa*, *P *=* *5.05 × 10^−39^, 86% vs. 68% in *A. lyrata*, *P *=* *3.73 × 10^−21^, χ^2^ test). On the basis of this result, we speculated that a high retention rate for the low diversified duplicates might be due to a high retention rate for the whole-genome duplicates.

To address whether the retention bias for the high or low diversified duplicates applied to the whole-genome or tandem duplicates, we compared the retention rates of the low diversified duplicates among the whole-genome and tandem duplicates. Of the whole-genome duplicates, the retention rates were significantly higher for the low diversified duplicates than for the high diversified duplicates in both *B. rapa* and *A. lyrata* (fig. 4*B*, 37% vs. 21% in *B. rapa*, *P *=* *5.55 × 10^−12^; 62% vs. 26% in *A. lyrata*, *P *=* *2.08 × 10^−44^; χ^2^ test). These results indicate that the low diversified duplicates after a whole-genome duplication may contribute to the functional compensation in multiple lineages. In contrast, of the tandem duplicates, the retention rates were significantly higher for the high diversified duplicates than for the low diversified duplicates in both *B. rapa* and *A. lyrata* (fig. 4*B*, 5.1% vs. 1.4% in *B. rapa*, *P *=* *2.06 × 10^−4^; 15% vs. 5.5% in *A. lyrata*, *P *=* *3.88 × 10^−8^; χ^2^ test). Although many of the tandem duplicates were lost in both *B. rapa* and *A. lyrata*, the retained genes tended to undergo functionalization, implying they may be associated with functions required in multiple lineages. Thus, it is likely that high diversified duplicates derived from tandem duplication events contributed to the adaptive evolution of the Brassicaceae lineage. Taken together, different duplication mechanisms play distinct roles of functional diversity in plant evolution.

### Selection Pressures on High and Low Diversified Duplicates

The retention bias associated with the high and low diversified duplicates in *B. rapa* and *A. lyrata* lineages may be influenced by a bias in the selection pressures between the high and low diversified duplicates. To examine the selection pressures, we focused on 484 and 801 pairs of *A. thaliana* duplicates with orthologs in *B. rapa* and *A. lyrata*, respectively. We then generated a phylogenetic tree for each duplicate pair with orthologs in *B. rapa* and *A. lyrata* (fig. 4*C*). A phylogenetic tree revealed an ancestral sequence between *A. thaliana* and *A. lyrata* (AS1) and another ancestral sequence between *A. thaliana* and *B. rapa* (AS2). As an indicator of selection pressures, we calculated the *K*_A_/*K*_S_ ratio for the two branches (AS1 for *A. lyrata* and AS2 for *B. rapa*). As control of selection pressure, we focused on 2,513 and 3,882 singleton orthologous groups keeping one-to-one relationship in *A. thaliana* to *B. rapa* and *A. thaliana* to *A. lyrata*, respectively (supplementary document 8, Supplementary Material online). As expected, low diversified orthologous duplicates tended to have a significantly lower *K*_A_/*K*_S_ ratio than the singleton orthologous genes in both branches (fig. 4*D*;*P *=* *2.97 × 10^−41^ [whole-genome duplicates in AS1-Al], *P *=* *4.79 × 10^−2^ [tandem duplicates in AS1-Al], *P *=* *1.33 × 10^−49^ [whole-genome duplicates in AS2-Br], and *P *=* *9.62 × 10^−3^ [tandem duplicates in AS2-Br], two-tailed Wilcoxon rank sum test). Additionally, high diversified orthologous duplicates tended to have a significantly higher *K*_A_/*K*_S_ ratio than singleton orthologous genes (fig. 4*D*;*P *=* *2.69 × 10^−11^ [whole-genome duplicates in AS1-Al], *P *=* *1.87 × 10^−8^ [tandem duplicates in AS1-Al], *P *=* *9.22 × 10^−28^ [whole-genome duplicates in AS2-Br] and *P *=* *3.68 × 10^−14^ [tandem duplicates in AS2-Br], two-tailed Wilcoxon rank sum test). These results indicate that high and low diversified duplicates assigned in *A. thaliana*, regardless of duplication mechanisms, tend to hold the same trend in closely related species.

To further clarify the trends in the natural selection of retained duplicates via functional divergence or duplication mechanisms, we searched for positive and purifying selections among the retained *B. rapa* and *A. lyrata* orthologs (supplementary document 9, Supplementary Material online). We also compared the proportions of the positive or purifying selection for the retained orthologs. Only high diversified duplicates resulting from tandem duplications had a higher proportion of positive selection than the average proportion for all genes in both *B. rapa* and *A. lyrata* (fig. 4*E*, *P* = 2.41 × 10^−2^ in AS2-Br and 2.74 × 10^−2^ in AS1-Al; χ^2^ test). Regarding the purifying selection, only low diversified duplicates due to whole-genome duplications had a higher proportion of purifying selection than the average proportion for all genes in both *B. rapa* and *A. lyrata* (fig. 4*F*, *P *=* *2.11 × 10^−2^ in AS2-Br and 5.66 × 10^−3^ in AS1-Al; χ^2^ test). An analysis of 100 bootstrapped data sets revealed that more than 95% of them supported the trends (all *P* values are listed in supplementary table S9, Supplementary Material online). These results suggest that high diversified duplicates due to tandem duplications tend to be under positive selection and that low diversified duplicates resulting from whole-genome duplications tend to be under purifying selection.

### Concluding Remarks

The copied genes immediately after a gene duplication event retain the original functions. During evolution, some of the duplicates undergo functionalization. In the present study, we developed a prediction method to infer high and low diversified duplicates based on the protein sequence divergence rate and the gene expression similarity rate. The classification of duplicates by our prediction model was validated via experimental and computational approaches. Furthermore, functional biases between high and low diversified duplicates were revealed based on GO terms, PPIs, broad or specific expression patterns, and duplication mechanisms, indicating that integrating these data in a model may be useful for predicting the high and low diversified duplicates.

Of 4,017 pairs of recently duplicated genes in *A. thaliana*, 1,052 (26.1%) and 600 (14.9%) were classified as the high and low diversified duplicates, respectively. We also determined that the classification tended to be associated with lineage-specific functions (e.g., related to biotic stress responses) and essential signaling pathways, respectively. Surprisingly, we detected considerably more high diversified duplicates than low diversified duplicates, implying that many lineage-specific functions may be due to gene duplication events. Although high diversified duplicates tended to be lost in closely related species under relaxed selection pressures, high diversified duplicates via tandem duplication tended to be retained by positive selection. Thus, as expected, gene duplication events extensively contribute to lineage-specific functions. low diversified duplicates, especially in whole-genome duplicates, tend to be retained in closely related species with purifying selection, though the number of low diversified gene pairs tends to be less than that of high diversified gene pairs. Whole-genome duplications influence functional compensation by substantially enhancing essential signaling pathways. Overall, the data presented herein indicate that duplicate genes have distinct functions depending on the functional divergence and duplication mechanisms.

## Materials and Methods

### Training Data for High and Low Diversified Gene Pairs

To improve the training data, we added 1,149 duplicate pairs for which the single or double knock-out mutant exhibited abnormal phenotypes in an earlier study (Lloyd and Meinke 2012) as well as data regarding polymorphisms and phenotypes data available in The Arabidopsis Information Resource (TAIR) database (version 10) (https://www.arabidopsis.org, last accessed Octobor 16, 2020). Thus, the training data comprised 1,275 and 366 possible pairs of high and low diversified genes, respectively. We compiled the nucleotide sequence and the encoded amino acid sequence of *A. thaliana* protein-coding genes from the TAIR database. For 1,641 duplicate pairs, the longest amino acid sequence encoded by a gene was used as a representative protein sequence and we aligned the sequences with the default settings of the MAFFT program (version 7.215) (Katoh and Standley 2013). We removed 531 duplicate pairs because their sequence identity was less than 30% and their sequence coverage was less than 50%. After adjusting for the insertion or deletion sites based on the aligned amino acid sequences, we generated the nucleotide sequences of the protein-coding regions. Regarding the aligned coding sequences, the *K*_S_ (i.e., number of synonymous substitutions per synonymous site) of 1,110 duplicate pairs was calculated with the yn00 program of PAML (version 3.14) (Yang 2007). Of the 1,110 duplicate pairs, 536 with *K*_S_ > 3 were removed from our training data. The remaining 574 duplicate pairs are listed in supplementary table S1, Supplementary Material online.

### Construction of a Model for Predicting the Functional Divergence of Duplicates

To predict the DFD of duplicates, we used three models which are based on LR, RF, and SVM algorithms (Agresti 2002; Byvatov and Schneider 2003; Díaz-Uriarte and Alvarez de Andrés 2006). For the three algorithms, we used 0 and 1 represented high and low diversified duplicates, respectively, as response variables. In three models, we used *K*_A_/*K*_S_ and Re/*K*_S_ as explanatory variables.

In LR analysis, coefficients were searched by conducting generalized linear model using the following setting (link = logit, family = binomial). The best formula by LR analysis was as follows: logit (DFD) = −0.1228 + 10.3044*K*_A_/*K*_S_−1.4177Re/*K*_S_, where DFD is the DFD. In RF analysis, the six parameters which should be set for conducting prediction were set as follow (Criterion = entropy, Minimal size for split = 10, Minimal child weight = 1, Maximal depth = 20, number of tree = 500, subset ratio = −1). To conduct the prediction of SVM, the seven parameters were set as a result of parameter tuning by performing ten cross-validation tests (type = C-classification, kernel = radial, *C* = 10, degree = 3, gamma = 1.0, cache size = 80, epsilon = 0.01). LR, RF, and SVM analyses were conducted under R software environment (Team R Development Core 2013).

To compare the performance of models generated by LR, RF, and SVM algorithms, we calculated the AUC-ROC and AU-PRC values among the best models (Robin et al. 2011). The AUC-ROC and AU-PRC values based on random sampling and perfect inference are 0.5 and 1, respectively. The AUC-ROC and AU-PRC values of three prediction models were 0.86 and 0.96 (LR), 0.82 and 0.94 (RF), and 0.82 and 0.92 (SVM), respectively (fig. 1*D* and *E*), indicating that the LR algorithm gave the best fit model for predicting high or low diversified duplicates. This AUC-ROC value was better than the reported moderately good value of 0.70 (Cholongitas et al. 2006).

DFD value where the false-positive rate was less than 5% were chosen as the thresholds of the classification of high and low diversified genes. As a result, the thresholds were the bottom 5% of the inferred DFD values were <0.54 (i.e., low DFD at 5% false-positive rate) and the top 5% of DFD values were >0.91 (i.e., high DFD at 5% false-positive rate), respectively (fig. 1*F* and *G*). These thresholds were also inferred by performing 100 cross-validation tests (supplementary document 10, Supplementary Material online), indicating that the false-positive rate using the thresholds is robust for predicting unknown data.

### Generation of Knock-Down Transgenic Plants and Evaluation of Knock-Down Effects

For the eight genes of the four duplicate pairs, we generated knock-down transgenic plants overexpressing artificial pre-miRNA sequences (Duan et al. 2008). The miR159a-based pre-miRNAs included an endogenous 23-nt sequence for repressing target gene expression. The potential 23-nt fragments of the eight genes recognized by the miRNAs were examined with the siDirect software (version 2.0; Naito 2009). To knock down individual duplicates, we selected gene-specific 23-nt sequences. To simultaneously knock down a duplicate pair, we selected a 23-nt sequence common to the duplicate pair. By replacing the original 23-nt sequence in miR159a, we generated eight and two artificial pre-miRNA sequences for knocking down eight single genes and two duplicate pairs, respectively (supplementary table S10, Supplementary Material online). The ten artificial pre-miRNA sequences were synthesized by Thermo Fisher Scientific (https://www.thermofisher.com, last accessed September 2, 2018). The synthesized DNA sequences were inserted into the pBST1 binary vector (Matsushita 2011) with the In-Fusion enzyme (Clontech, Heidelberg, Germany).

The recombinant binary vector was subsequently introduced into Agrobacterium tumefaciens (strain GV3101) cells and then inserted into the A. thaliana genome according to the floral-dip method (Clough and Bent 1998). After obtaining plants from three independent T1 plants, we examined the phenotypes of the transgenic T2 generation plants of in plates containing agar-solidified Murashige and Skoog medium (Sigma–Aldrich, Taufkirchen, Germany) supplemented with Basta (10 mg/l). Transgenic plants were grown vertically on the same plate under continuous light at 22 °C for 10 days. The phenotypic analyses were completed with at least three independent lines and at least three biological replicates (supplementary fig. S5, Supplementary Material online). Additionally, we measured the hypocotyl and root lengths with the ImageJ software for ten biological replicates (Schneider et al. 2012).

## Supplementary Material

Supplementary data are available at *Molecular Biology and Evolution* online.

## Supplementary Material

msaa302_Supplementary_Data
