# Supplementary material for: Degree of Functional Divergence in Duplicates Is Associated with Distinct Roles in Plant Evolution
Source: Mol Biol Evol. 2020 Dec 8;38(4):1447–59. doi: 10.1093/molbev/msaa302 (PMC8042753; doi:10.1093/molbev/msaa302)
Supplement: msaa302_Supplementary_Data [file msaa302_supplementary_data.zip › Supple_Figs.pdf]

Fig. S1

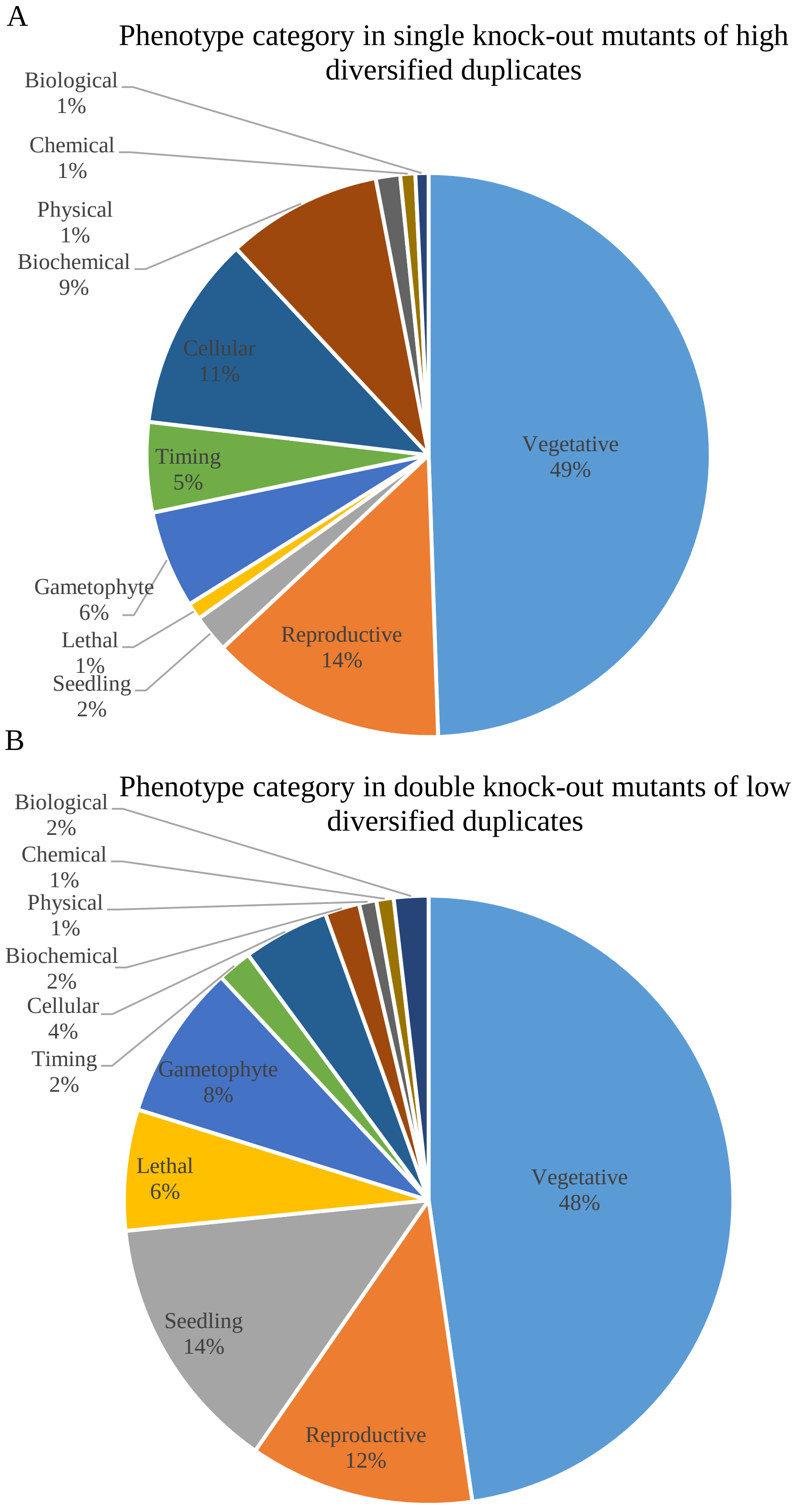

Fig. S1. Phenotypic categories in single and double knock-out mutants. Pie charts show the phenotypic categories of single (A) and double knock-out mutants (B) assigned as high and low diversified duplicates in the training data, respectively. The phenotypic categories were based on previously described categories (Hanada 2009; Lloyd 2012).

Fig. S2

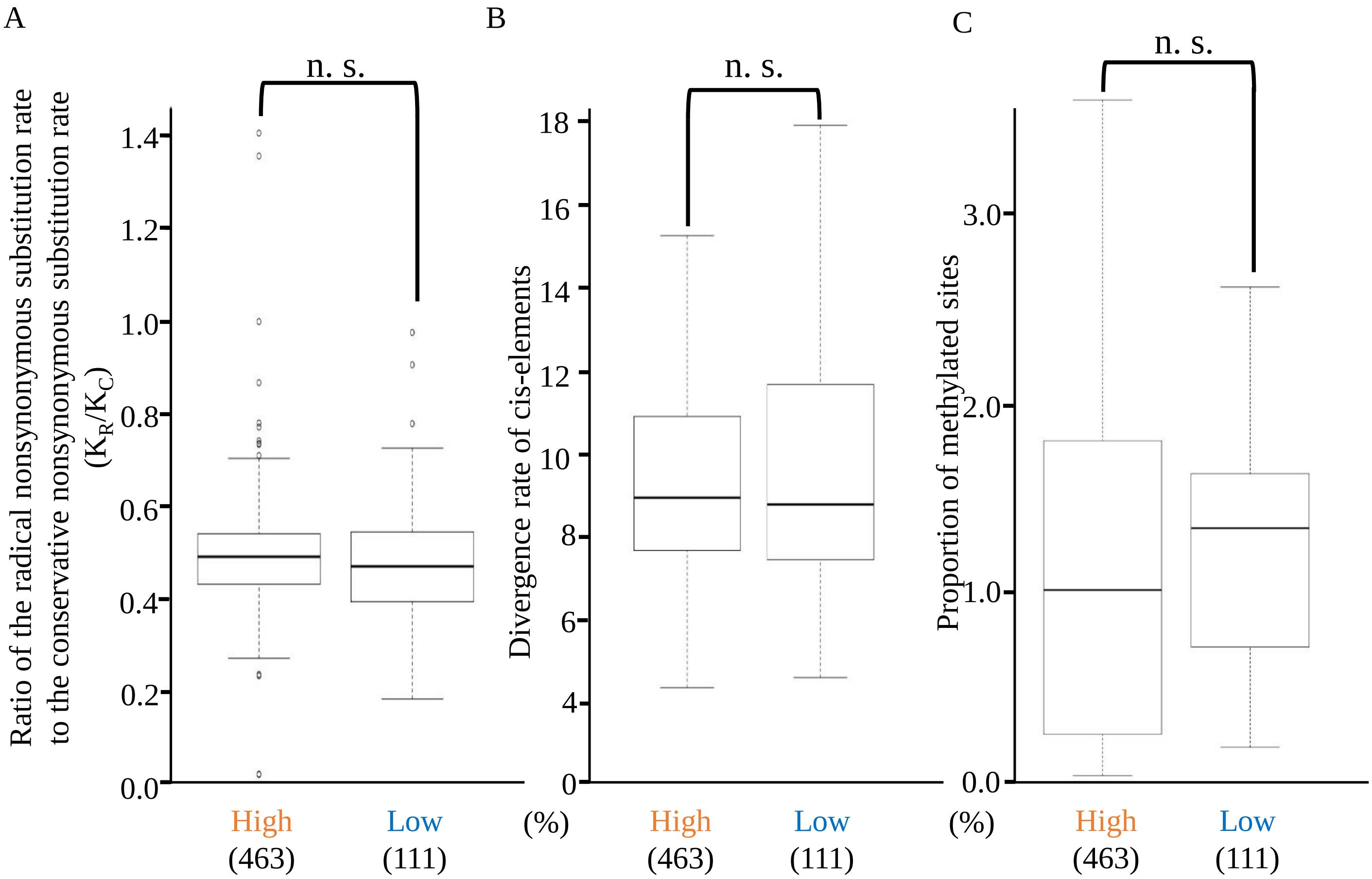

Fig. S2. Divergence rate of protein sequences, cis-elements and methylation sites in high and low diversified duplicates. (A) As a degree of divergence rate of protein sequences in duplicate pairs, the ratios between K_R (the number of chemically radical replacements per radical amino acid site) and K_C (the number of chemically conservative replacements per conservative amino acid site) were calculated in high and low diversified duplicates. Numbers in parenthesis represent the number of duplicate pairs. (B) The divergence rate of cis-elements represents the total number of observed cis-elements divided by the number of shared cis-elements in the duplicate pair. (C) As another similarity rate of gene expression in duplication pairs, the proportion of methylated sites were determined in high and low diversified duplicates. In box plots, the solid horizontal line represents the median value. The boxes and dotted lines represent the inter-quartile range (25%–75%) and he first to the 99th percentile. The n. s. indicates no significant difference ($P > 0.05$, two-tailed Wilcoxon rank sum test).

Fig. S3

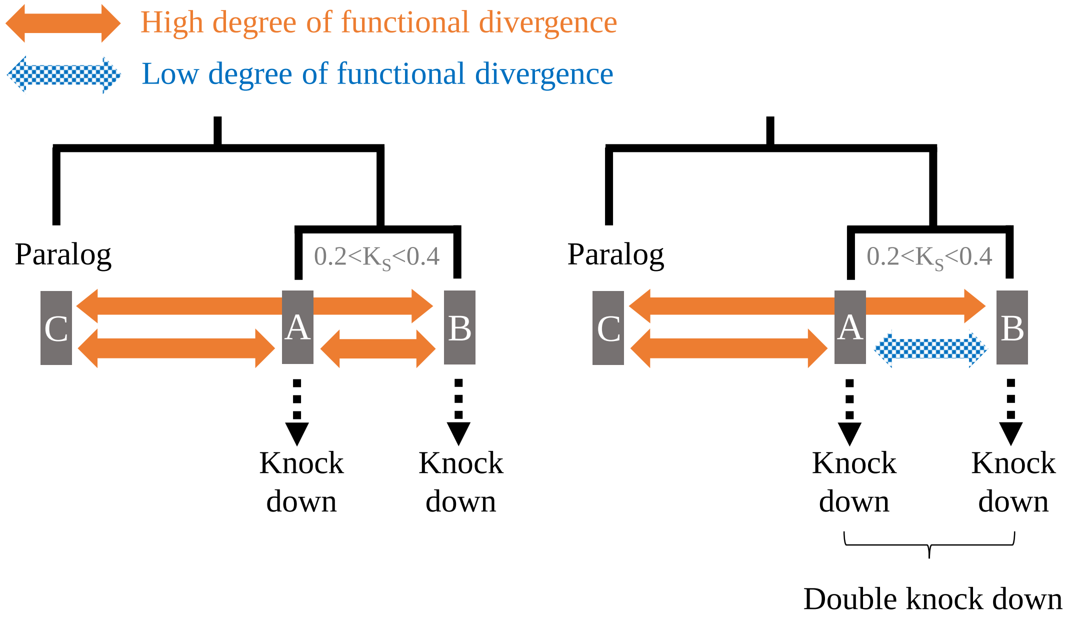

Fig. S3. Selection of target genes to be knocked down. A duplicate pair and the second closest paralog are presented in phylogenetic trees. The K_S (number of synonymous substitutions per synonymous site) should be > 0.2 and < 0.4 for the targeted duplicate pairs.

Fig. S4

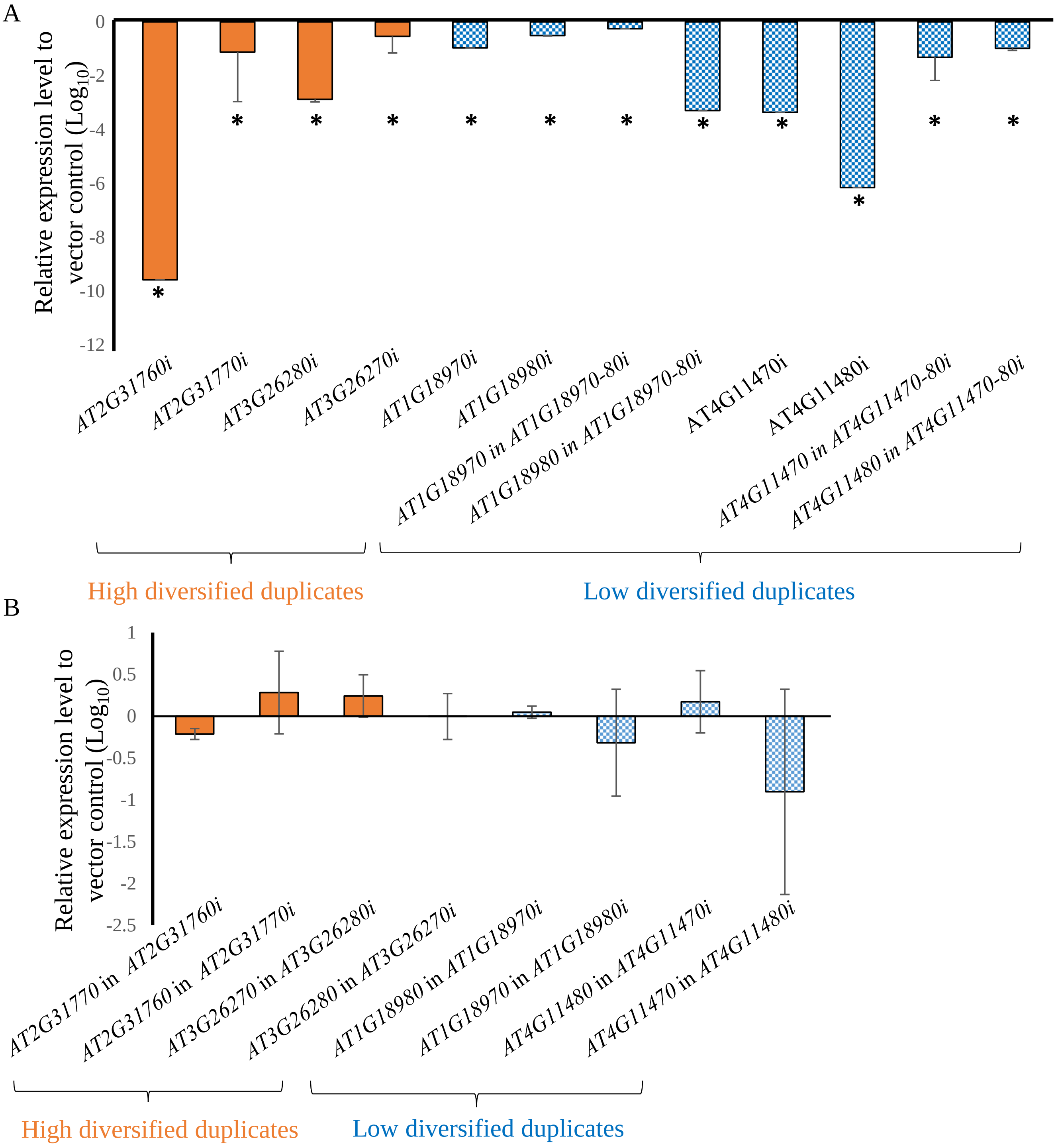

Fig. S4. Relative expression levels of knock-down transgenic plants to vector control plants. (A) Relative expression levels of targeted genes in knock-down transgenic plants. (B) Relative expression levels of off-targeted genes in knock-down transgenic plants. The bars with solid and checked pattern represent the relative expression levels of high and low diversified duplicates in the knock-down transgenic plants, respectively. The asterisk indicates a significant difference of the expression levels compared with the vector control (FDR < 0.05, two-tailed Wilcoxon rank sum test). The relative expression levels to the vector control and FDR are shown in S4 Table.

Fig. S5

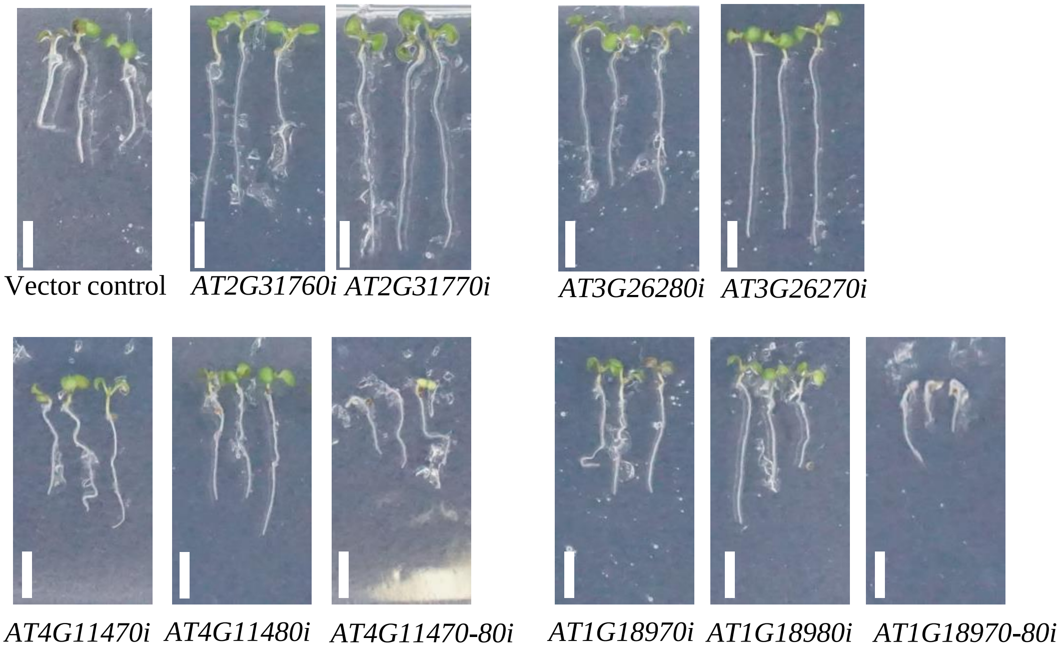

Fig. S5. Phenotypes of knock-down mutants and vector control plants. The white bars represent 1 cm. Three plants shown in a box were biological replicates generated from independent T_1 transgenic plants. Transgenic plants with a single gene knocked down (AT2G31760, AT2G31770, AT3G26270, AT3G26280, AT1G18970, AT1G18980, AT4G11470 and AT4G11480) are indicated by AT2G31760i, AT2G31770i, AT3G26270i, AT3G26280i, AT1G18970i, AT1G18980i, AT4G11470i, and AT4G11480i, respectively. Transgenic plants with double knocked down (AT1G18970 and AT1G18980 as well as AT4G11470 and AT4G11480) are indicated by AT1G18970-80i and AT4G11470-80i, respectively.

Fig. S6

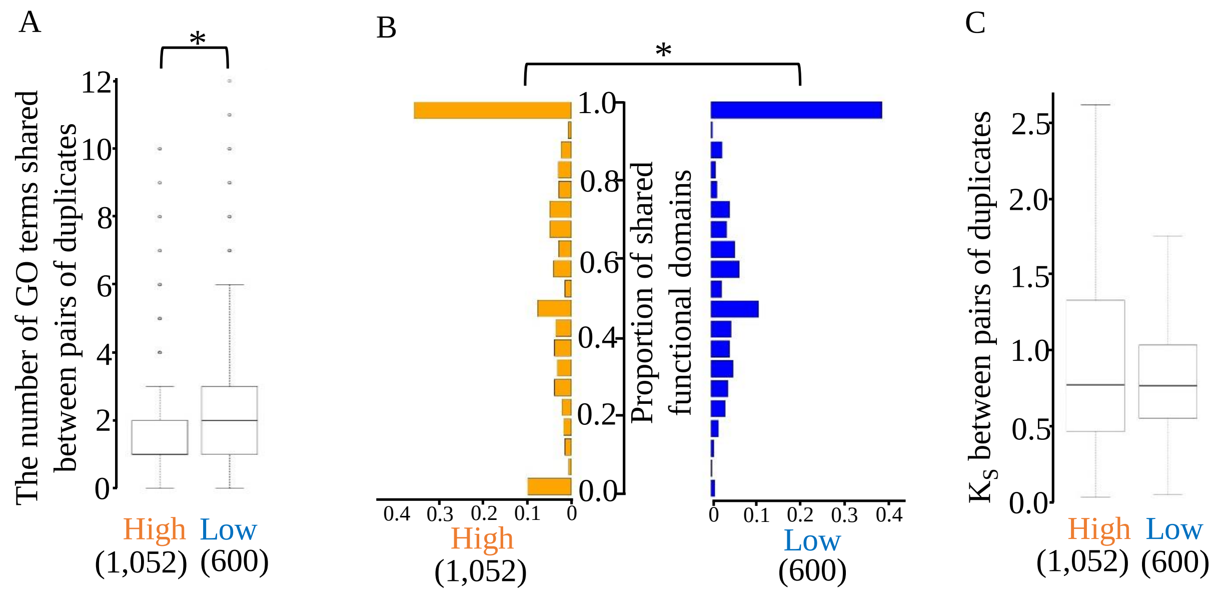

Fig. S6. Number of shared Gene Ontology (GO) terms, shared functional domains and K_S values in high and low diversified duplicates. (A) The distributions of the number of GO terms shared in duplicate pairs are presented in box plots with the solid horizontal line indicating the median value, the box representing the inter-quartile range (25–75%), and the dotted lines indicating the first to the 99th percentile. Genes that were not annotated with GO terms were excluded. The asterisk indicates a significant difference ($P < 0.05$, two-tailed Wilcoxon rank sum test). Numbers in parenthesis represent the number of duplicate pairs. (B) Histograms of proportion of shared functional domains between duplicates. Orange and blue bars represent high and low diversified duplicates, respectively. (C) K_S values in duplicate pairs are presented in box plots with the same manner.

Fig. S7

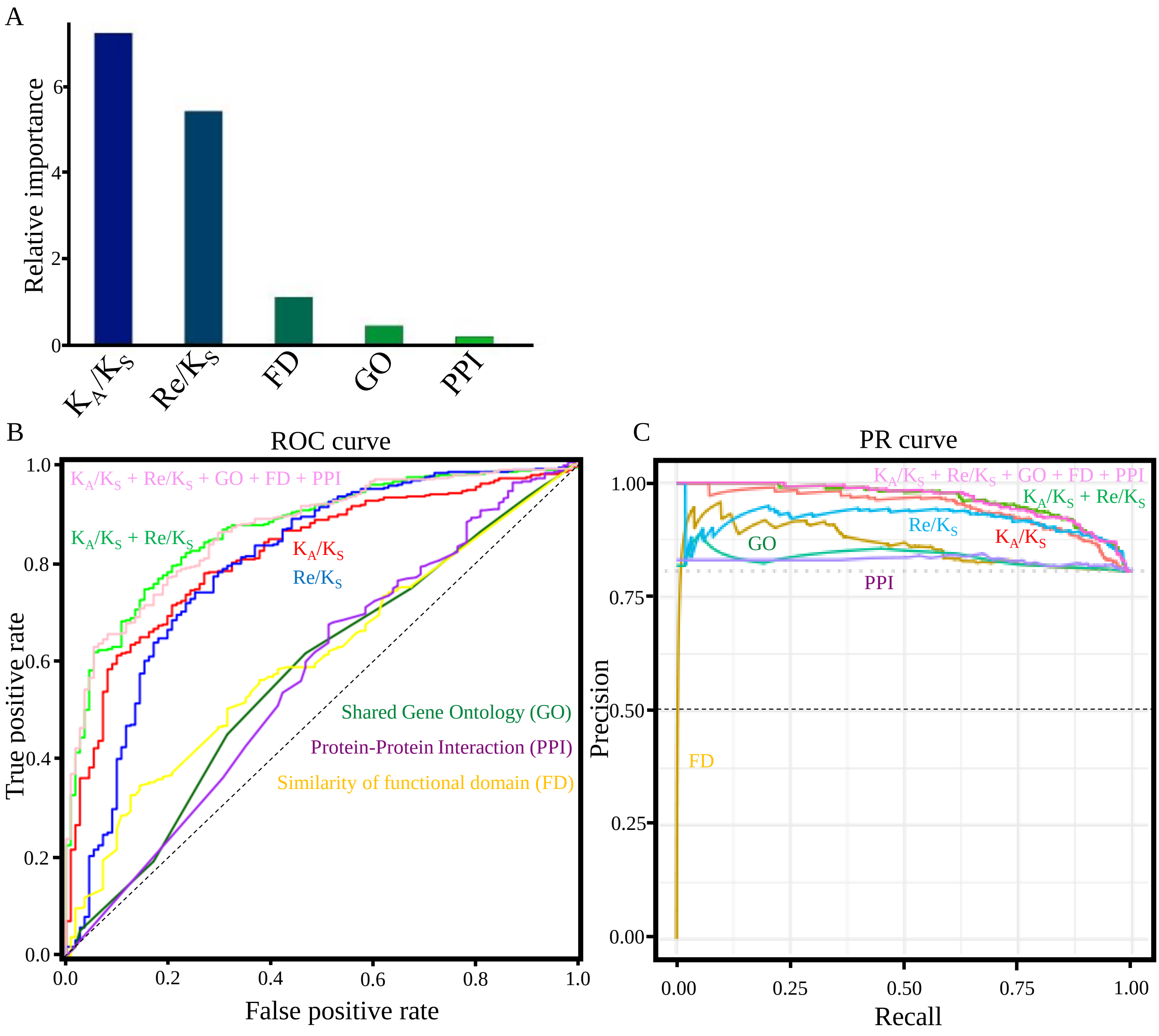

Fig. S7. Receiver Operating Characteristic (ROC) curve, Precision-Recall Curve (PRC) and relative importance of the parameters. (A) Relative importance in explanatory variables. The relative importance was inferred based on the logistic regression algorithm. (B,C) Three and seven colored lines represent ROC or PRC curves for each model. Dotted lines indicates that Area Under the Curve (AUC) – ROC or Area Under the PRC (AU-PRC) values were 0.5, which indicates the performance of a model classifying at random.

Fig. S8

A

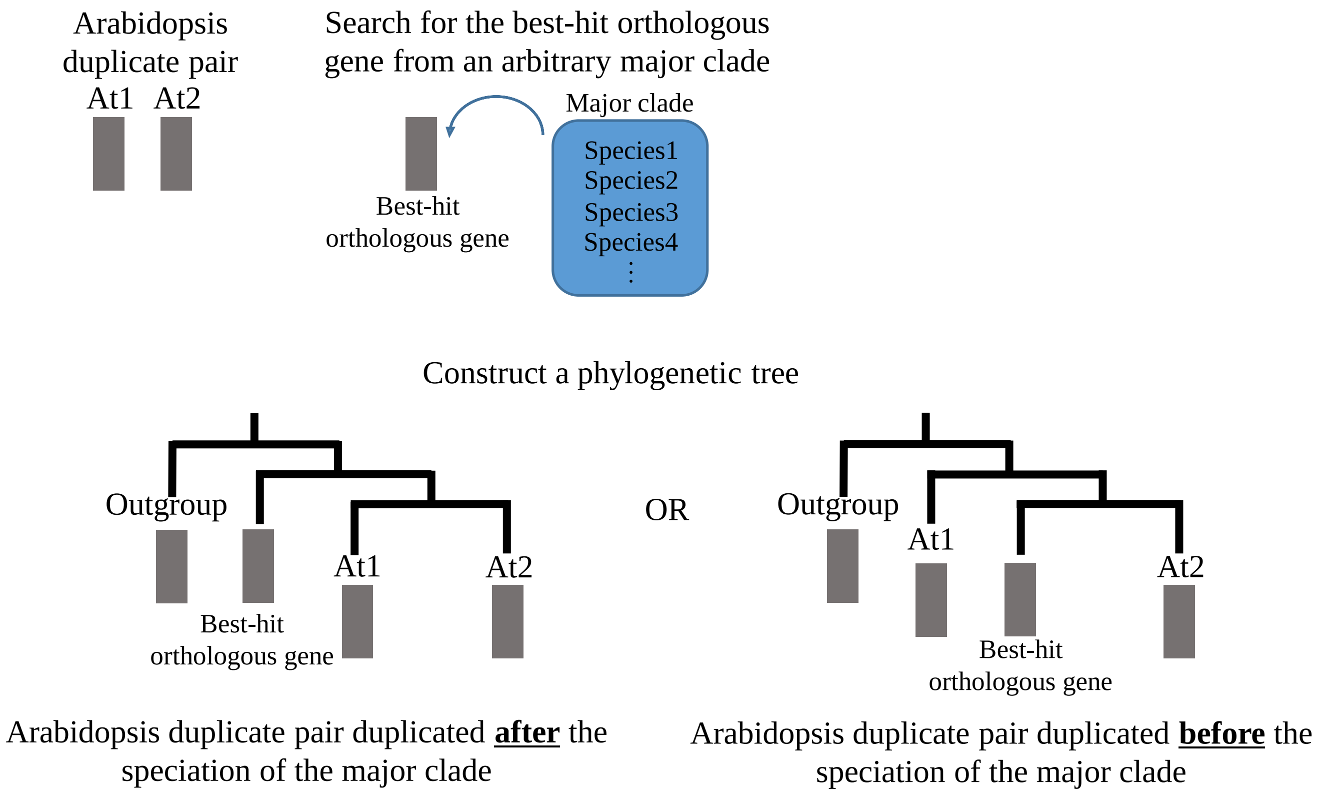
Arabidopsis
duplicate pair
At1 At2
Search for the best-hit orthologous
gene from an arbitrary major clade
Major clade
Species1
Species2
Species3
Species4
⋮
Best-hit
orthologous gene
Construct a phylogenetic tree
Outgroup
Best-hit
orthologous gene
At1
At2
OR
Outgroup
At1
Best-hit
orthologous gene
At2
Arabidopsis duplicate pair duplicated after the
speciation of the major clade
Arabidopsis duplicate pair duplicated before the
speciation of the major clade

B

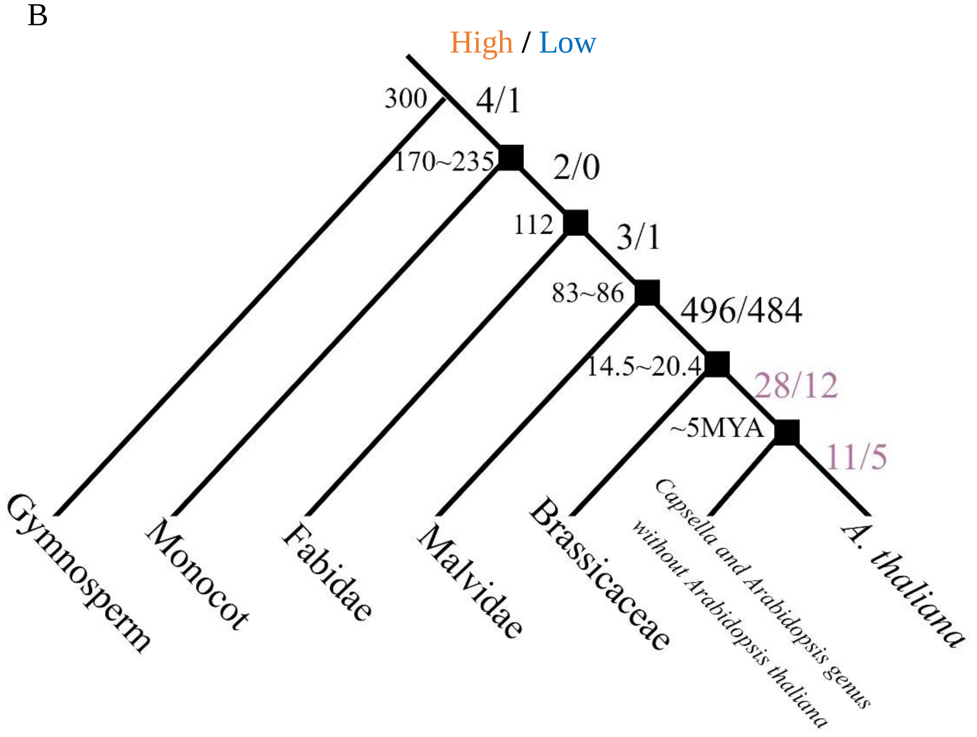
High / Low
300
4/1
170~235
2/0
112
3/1
83~86
496/484
14.5~20.4
28/12
~5MYA
11/5
Gymnosperm
Monocot
Fabidae
Malvidae
Brassicaceae
Capsella and Arabidopsis genus
without Arabidopsis thaliana
A. thaliana

Fig. S8. Estimation of the timing of duplication events. (A) Methodology for estimating the duplication times. We performed a similarity search of two paired *A. thaliana* duplicates (At1 and At2) against all proteins of 32 plant species with BLASTP (version 2.2.6). The best-hit orthologous gene in a pair was identified in each of eight clades, including CAG, BRA, MAL, FAM, AST, MONO, and GYM (S7 Table). The best-hit genes in GYM were used as outgroups. To examine the duplication time for an *A. thaliana* duplicate pair based on the topology of phylogenetic trees, the duplication and speciation timings were compared between *A. thaliana* and CAG, BRA, FAM, AST, or MONO. If the topology of the phylogenetic tree indicated that the duplication event for At1 and At2 occurred after the branching from the best-hit orthologous gene, the *A. thaliana* duplicate pairs were considered to have duplicated after the speciation of the major clade. If the topology of the phylogenetic tree indicated that the duplication event for At1 and At2 occurred before the branching from the best-hit orthologous gene, the *A. thaliana* duplicate pairs were considered to have duplicated before the speciation of the major clade. (B) The seven major clades of a species tree are presented. The branch length does not provide any information. The numbers to the left of nodes represent the time from speciation (million years ago; MYA). The two numbers to the right of branches represent the number of duplicate pairs with high and low diversified duplicates that resulted from a duplication event at the indicated time. The duplicates presented in pink were excluded from the analysis of retention rates because of the possibility these lineage-specific duplicated genes were lost.
