# Supplementary material for: Degree of Functional Divergence in Duplicates Is Associated with Distinct Roles in Plant Evolution
Source: Mol Biol Evol. 2020 Dec 8;38(4):1447–59. doi: 10.1093/molbev/msaa302 (PMC8042753; doi:10.1093/molbev/msaa302)
Supplement: msaa302_Supplementary_Data [file msaa302_supplementary_data.zip › Supplemental_document_ClearCopy.pdf]

Supplemental document 1: Divergence rate of the encoded protein sequences, gene expression similarity rate, cis-elements and methylation in promoter regions of duplicate pairs.

We calculated the K_A (the number of nonsynonymous substitutions per nonsynonymous site) and K_S (the number of synonymous substitutions per synonymous site) of 574 duplicate pairs with the yn00 program of PAML (Yang and Nielsen 2000; Yang 2007). We also calculated the K_R (the number of chemically radical replacements per radical amino acid site) and K_C (the number of chemically conservative replacements per conservative amino acid site) of 574 duplicate pairs based on amino acid classification that Hanada et al. generated (Hanada et al. 2006). To infer the functional divergence of duplicate pairs, we used K_A/K_S and K_R/K_C as explanatory variables of protein divergence.

The degree of the similarity in the expression of 574 duplicate pairs was examined based on the microarray data of the AtGenExpress expression atlas in the TAIR database. The microarray data for 20,151 genes represented 634 conditions, comprising 82 developmental stages, 72 biotic treatments, 285 abiotic treatments, 11 nutrient treatments, 81 hormone treatments, 40 chemical treatments, 21 cell cycle stages, and 42 genotypes. The expression levels in the microarray were processed with the Bioconductor (http://www.bioconductor.org) affy package in the R software environment (Team RC 2013). The similarity of expression patterns (Re) in a duplicate pair was determined based on Spearman’s correlation coefficient for the expression levels of the pair among 634 conditions. However, Re was strongly influenced by the duplication age (sequence divergence) (Hanada et al. 2009; Hanada et al. 2010). To minimize the effect of the duplication age, Re was divided by K_S.

As the other factors of expression divergence, we collected cis-elements in 1 kb promoter regions (1kbp upstream from either start codon or transcriptional start site) of each of

annotated *A. thaliana* genes in TAIR10. To examine the divergence of cis-elements in each duplicate pair, we calculated the proportion of overlapping cis-elements which is the number of shared cis-elements in duplicate pair. The divergence rate of cis-elements was defined to be the total number of observed cis-elements divided by the number of shared cis-elements in the duplicate pair. We also collected methylated cytosine sites in 1 kb promoter regions of each of annotated *A. thaliana* genes based on the reference genome (Col-0) in 1001 Arabidopsis Genomes, Methylomes, Transcriptomes and Physical Maps (http://neomorph.salk.edu, GSM1085222) (Kawakatsu et al. 2016). We calculated the proportion of methylated cytosine sites in the promoter regions of all annotated *A. thaliana* genes.

To infer the functional divergence of duplicate pairs, we used only K_A/K_S and Re/ K_S as explanatory variables because K_A/K_S and Re/ K_S are highly associated with the functional divergence. However, K_R/K_C is not associated with the functional divergence because K_R/K_C tends not to be different between high and low diversified duplicates (**Fig. S2A**; P = 0.10, by the two-tailed Wilcoxon rank sum test). Additionally, the proportions of overlapping cis-elements and methylated cytosine sites tend not to be different between high and low diversified duplicates as well (**Fig. S2B**; P = 0.06 in cis-elements, **Fig. S2C**; P = 0.52 in methylated cytosine sites, two-tailed Wilcoxon rank sum test). Therefore, we did not use K_R/K_C, cis-elements and methylated cytosine sites as explanatory variables to generate the mathematical model of functional divergence. The all parameters that we tested were listed in **S1 Table**.

Supplemental document 2: Inferring high and low diversified genes among *Arabidopsis thaliana* duplicates.

We compiled the nucleotide sequence and the encoded protein sequence of 27,416 annotated protein coding genes in the TAIR database. The longest protein sequence encoded by

a gene was used as a representative sequence. The genes were used as queries to search for homologous *A. thaliana* genes with BLASTP (version 2.2.6) (E-value = 1.00×10^{-4}) (Altschul 1997). After focusing on the duplicate pairs with the best hits, we aligned the sequence pairs with the default settings of MAFFT (version 7.215) (Katoh and Standley 2013). If the sequence identity of the duplicate pair was less than 30% and the sequence coverage was less than 50%, the duplicate pair was not considered for further analyses. Accordingly, we identified 4,017 pairs of recently duplicated genes in *A. thaliana*, of which 1,052 and 600 pairs were predicted to be high and low diversified duplicate pairs, respectively.

Supplemental document 3: Validation of four duplicate pairs.

To validate the degree of functional divergence in duplicate pairs, we examined the phenotypic changes due to knocking down two high diversified pairs and two low diversified pairs. To the best of our knowledge, there was a lack of available information regarding the phenotypes associated with the eight genes of the four selected pairs. Additionally, the four gene pairs should have undergone functional diversification before the gene duplication event (**Fig. S3**). This is because knocking down the selected duplicates was not expected to lead to phenotypic changes if the second closest paralog has functions redundant to those of the selected duplicates. Of the 1,652 duplicate pairs that were designated as 1052 high and 600 low diversified pairs, we tried to identify a second closest paralogs against a duplication pair by performing similarity searches with BLASTP (version 2.2.6) (Altschul 1997). Out of 1,652 pairs, only 507 pairs had the same second closest paralog within the following criteria (identity>30%, coverage>50%, and K_S<3). In 507 pairs, we re-examined the functional divergence between each duplicate pair and the second closest paralogs with our model, and identified 44 pairs that were predicted to be high diversified. Out of the 44 pairs, we removed 28 pairs that shared the same second closest paralogs

with the other duplicate pairs. Remaining 16 pairs were designated as 6 high and 10 low diversified duplicates. We examined whether selected 16 pairs were highly expressed in initial growth of seedlings or not because our phenotypic analyses involved initial growth. There are 165 expression profiles at seeding stages in the microarray data (S2 Table). In the case that a gene is expressed in more than 100 expression intensities at least two experiments of seedling stages, the gene is defined to be expressed in seedling stage. Out of 16 pairs, 12 pairs had more than 100 expression intensity under the conditions. Among 12 pairs, 4 and 8 pairs of high and low diversified pairs. To decrease the bias associated with the duplication mechanism and timing, we focused on the tandemly duplicated genes and young duplicates with $0.2 < K_S < 0.4$. We finally identified two pairs of young and tandemly duplicated genes that had undergone a functionalization and two pairs of young and tandemly duplicated genes that were functionally redundant.

Supplemental document 4: qRT-PCR experiments in knock-down transgenic plants.

Total RNA was extracted from three independent T_2 lines of transgenic plants using Plant RNA purification reagent (Invitrogen, Massachusetts, USA). Complementary DNA was synthesized using a Quantitect Reverse Transcription Kit (Qiagen, Hilden, Germany) according to the manufacturer's instructions. All RNA samples were adjusted to the same concentration. cDNA was synthesized from 7 μg of total RNA using an oligo(dT) primer and QuantiTect Rev. Transcription Kit (QIAGEN, Hilden, Germany). Real-time PCR was performed using the protocol for the Mx3000P qPCR System (Agilent Technologies, California, USA). The PCR analyses were performed using THUNDERBIRD SYBR qPCR Mix (TOYOBO, Osaka, Japan) and the products were analyzed using the Mx3000P multiplex quantitative PCR system (Agilent Technologies, California, USA). Gene transcript levels were normalized against that of TUB2.

The primer sets used are shown in **S3 Table**. The relative expression levels in the transgenic plants were compared with vector control by two-tailed Wilcoxon rank sum test. To correct for multiple testing, the false discovery rate (FDR) was estimated with the R-library Q-VALUE software. The null hypothesis that the two ratios are derived from the same population was rejected if the FDR was less than 0.05.

Supplemental document 5: Computational validations using functional annotations with Gene Ontology terms and functional domain information.

In total, 25,559 Gene Ontology (GO) terms are assigned in *A. thaliana* genes in the TAIR database. Of three main GO categories (cellular components, molecular functions, and biological processes), we only analyzed biological processes. The biological process GO terms were hierarchically assigned. The *A. thaliana* genes assigned a GO term were also assigned all of the corresponding parent GO terms. Among 25,559 GO terms, 648 and 579 GO terms were assigned to high and low diversified duplicates, respectively. We also collected functional domains identified by FPrintScan from TAIR10 (Scordis et al. 1999). We then examined shared GO terms and shared functional domains in the duplicate pairs.

To examine the enrichment of GO terms associated with the high and low diversified duplicates, the expected ratio was calculated as the ratio between the number of genes assigned to the GO term and the number of genes not assigned to the GO term among all annotated *A. thaliana* genes. For each GO term, the observed ratio for the genes with high and low diversified duplicates was compared with the expected ratio by a chi-squared test in the R software environment (Team RC 2013). To correct for multiple testing, the false discovery rate (FDR) was estimated with the R-library Q-VALUE software. The null hypothesis that the two ratios are derived from the same population was rejected if the FDR was less than 0.05.

Supplemental document 6: Enrichment analysis of core genes, protein-protein interactions, tandem/whole-genome duplicates, and specifically/broadly expressed genes.

A core gene set was obtained with CEGMA (version 2.5) (Parra et al. 2007). The protein-protein interaction data were obtained from the TAIR database, but we focused on the experimentally validated protein-protein interaction data. Tandemly duplicates and whole-genome duplicates were obtained from the preceding studies (Bowers et al. 2003; Shirai et al. 2017). To identify specifically and broadly expressed genes, we analyzed the microarray data of the AtGenExpress expression atlas covering 634 conditions. Median expression levels were calculated for each condition. The genes with expression levels higher than the top 75% of expression level in more than 95% (602 conditions) of the analyzed conditions were considered to be broadly expressed. The specifically expressed genes were those with expression levels that were higher than the top 25% of expression levels in at least one condition and those with expression levels that were lower than the bottom 25% of expression levels in more than 95% (602) of the analyzed conditions. Accordingly, we identified 2,001 specifically and 115 broadly expressed genes. Expression data about genes that we used in this manuscript is accessible online (http://labo.bio.kyutech.ac.jp/~kohanada/).

These variables [the number of shared GOs (GO), the number of shared functional domains (FD), Protein-Protein interaction (PPI), core genes and broadly expressed genes] seem to be useful to infer the functional divergence of duplicates. However, out of five variables, two variables (core genes and broadly expressed genes) are not assigned in most of duplicates. Focusing on three variables (GO, FD and PPI) as well as K_A/K_S and Re/K_S, we generated prediction models in each of variables (Figs. S7B-C). Furthermore, the coefficients were calculated based on the generalized linear model using the following formula: logit (DFD)=

$0.3921729 + 10.01193K_A/K_S - 1.405118Re/K_S - 0.0004153765PPI - 0.02401407GO - 0.5097127FD$ where DFD is the degree of functional divergence. The all parameters that we used were listed in **S1 Table**.

Supplemental document 7: Estimation of the timing of duplication events.

We compiled the nucleotide sequences of annotated protein coding genes from 33 plant species in the TAIR, Phytozome (version 10.1; http://www.phytozome.net/), and gymnosperm (http://congenie.org) databases (Nystedt et al. 2013). We classified 33 plant species into eight major clades: Gymnosperms (GYM), Monocots (MONO), Asterids (AST), Fabidae (FAB), Malvidae without Brassicaceae (MAL), Brassicaceae without Arabidopsis and Capsella (BRA), Capsella and Arabidopsis without *A. thaliana* (CAG), and *A. thaliana* (**S7 Table**).

We predicted 1,052 and 600 duplicate pairs with high and low diversified pairs, respectively. We performed a similarity search with 3,304 *A. thaliana* duplicates of 1,652 pairs against all proteins of 32 plant species with BLASTP (version 2.2.6) (E-value $< 1.00 \times 10^{-9}$) (Altschul 1997). For a duplicate pair, we identified the best-hit genes in each of the eight major clades. The best-hit genes in GYM were used as outgroups. To examine the duplication timing for an *A. thaliana* duplicate pair based on the topology of phylogenetic trees, we compared the duplication and the speciation timings between *A. thaliana* and CAG, BRA, FAM, AST, or MONO (**Fig. S8A**). For example, to compare the duplication and the speciation timings between *A. thaliana* and CAG, we aligned the duplicate pairs, the best-hit gene in CAG, and the best-hit gene in GYM with MAFFT (version 7.215) (Katoh and Standley 2013) and constructed phylogenetic trees according to the neighbor-joining method (Saitou and Nei 1987) of ClustalW (version 1.83) (Thompson et al. 2003). We re-examined the duplication timings in BRA, MAL,

FAM, AST, and MONO with the same procedure. We ultimately identified 1,047 duplication events in six evolutionary periods (**Fig. S8B**).

Supplemental document 8: Identification of orthologs of *A. thaliana* duplicates in *B. rapa* or *A. lyrata*.

Of 1,047 duplicate pairs, we focused on 991 duplicate pairs whose duplication events occurred before the divergence of the Brassica and Arabidopsis lineages. For 1,982 *A. thaliana* duplicates in 991 pairs, we performed a similarity search between *A. thaliana* and either *B. rapa* or *A. lyrata* with BLASTP (version 2.2.6) (Altschul 1997). The reciprocal best-hit pair (E-value $< 1.0 \times 10^{-9}$) was defined as the orthologous pair between *A. thaliana* and either *B. rapa* or *A. lyrata*. We detected 1,031 and 1,718 orthologous genes in *B. rapa* and *A. lyrata,* respectively. Additionally, 484 and 801 orthologous gene pairs in *B. rapa* and *A. lyrata*, respectively, were regarded as retained orthologous gene pairs.

To identify orthologous singletons between *A. thaliana* and *B. rapa* or *A. lyrata*, we focused on 2,380 *A. thaliana* singletons which have been confirmed by EST analyses in pATsi database (Ambrosino et al. 2016). The singletons in *B. rapa* and *A. lyrata* were defined in the case that we could not find any similar protein sequences by BLASTP (version 2.2.6, E-value $< 1.0 \times 10^{-4}$) (Altschul 1997). Out of 40,492 genes in *B. rapa* and 32,657 genes of *A. lyrata*, we identified 2,513 and 3,882 singletons, respectively. To identify the orthologs of singletons between *A. thaliana* and *B. rapa* or between *A. thaliana* and *A. lyrata*, we performed a similarity search between *A. thaliana* and either *B. rapa*, *A. lyrata* singleton genes with BLASTP. The reciprocal best-hit pairs (E-value $< 1.0 \times 10^{-9}$) were defined as the orthologous singleton pairs between *A. thaliana* and either *B. rapa*, or *A. lyrata*.

Supplemental document 9: Estimation of ancestral sequences and calculation of K_A/K_S.

Of 991 *A. thaliana* duplicate pairs, we selected 484 and 801 orthologous gene pairs that were retained in *B. rapa* and *A. lyrata,* respectively. For each duplicate pair with orthologous genes in *B. rapa* and *A. lyrata*, a multiple sequence alignment was prepared with the default parameters of MAFFT (version 7.215) (Katoh and Standley 2013). We predicted ancestral sequences (AS) between *A. thaliana* and *A. lyrata* or *B. rapa* with codeml (runmode = 0, model = 1, NSsites = 0) in PAML (version 3.14) (Yang 2007). To identify the ancestral sequences, we determined the optimal initial omega [K_A/K_S parameter in PAML (version 3.14)] between 0 and 2 (increments of 0.1). The initial omega with the highest likelihood value was selected. Moreover, the K_A and K_S were calculated with yn00 in PAML (version 3.14) (Yang and Nielsen 2000; Yang 2007) based on the ancestral sequences.

To elucidate the selection pressures, we independently estimated the K_A/K_S in two terminal branches (AS1 for *A. lyrata* and AS2 for *B. rapa*) using a model with K_A/K_S as a free parameter (runmode = −2, fix-omega = 0). The maximum likelihood values of the model were compared with those of a model with K_A/K_S fixed at 1 (runmode = −2, fix-omega = 1, omega = 1) according to the likelihood ratio test. The *P*-values were corrected based on the FDR with the R-library Q-VALUE software. The genes affected by positive selection were defined as those with an FDR < 0.05 and $K_A/K_S > 1$. Similarly, the genes affected by purifying selection were defined as those with an FDR < 0.05 and $K_A/K_S < 1$.

To evaluate whether the observed numbers of positive or purifying selection are higher than the expected numbers or not, we prepared 100 sets of 484 and 801 duplicate pairs whose sequences were sampled by bootstrap method. Based on the above procedure, we counted the number of genes affected by positive or purifying selection in two terminal branches in each of bootstrapped sets. The analyses were repeated 100 times. The results were shown in **S9 Table**.

Supplemental document 10: Cross validation test for inferring the threshold of the prediction model.

The thresholds for identifying high and low diversified genes with the formula were defined by performing 100 cross-validation tests. Five hundred seventy-four pairs of the training data were randomly split into 100 parts. The prediction model was trained based on the 99 parts, and the model predicted the one part. After this procedure was repeated 100 times, the thresholds where the false positive rate was under 5% was used. As a result, the cross-validation test supported that < 0.54 (i.e., low DFD at 5% false positive rate) and the top 5% of DFD values were > 0.91 (i.e., high DFD at 5% false positive rate) were robust thresholds for the prediction model.

References

Altschul S. 1997. Gapped blast and psi-blast: a new generation of protein database search programs. *Nucleic Acids Res.* 25:3389–3402.

Ambrosino L, Bostan H, di Salle P, Sangiovanni M, Vigilante A, Chiusano ML. 2016. PATsi: paralogs and singleton genes from arabidopsis thaliana. *Evol. Bioinforma.* 12:EBO.S32536.

Bowers JE, Chapman BA, Rong J, Paterson AH. 2003. Unravelling angiosperm genome evolution by phylogenetic analysis of chromosomal duplication events. *Nature* 422:433–438.

Hanada K, Gojobori T, Li W-H. 2006. Radical amino acid change versus positive selection in the evolution of viral envelope proteins. *Gene* 385:83–88.

Hanada K, Kuromori T, Myouga F, Toyoda T, Li WH, Shinozaki K. 2010. Evolutionary persistence of functional compensation by duplicate genes in arabidopsis. *Genome Biol. Evol.* 1:409–414.

Hanada K, Kuromori T, Myouga F, Toyoda T, Shinozaki K. 2009. Increased expression and

protein divergence in duplicate genes is associated with morphological diversification.Walsh B, editor. *PLoS Genet.* 5:e1000781.

Katoh K, Standley DM. 2013. MAFFT multiple sequence alignment software version 7: improvements in performance and usability. *Mol. Biol. Evol.* 30:772–780.

Kawakatsu T, Huang SC, Jupe F, Sasaki E, Schmitz RJ, Urich MA, Castanon R, Nery JR, Barragan C, He Y, et al. 2016. Epigenomic diversity in a global collection of arabidopsis thaliana accessions. *Cell* 166:492–505.

Nystedt B, Street NR, Wetterbom A, Zuccolo A, Lin Y-C, Scofield DG, Vezzi F, Delhomme N, Giacomello S, Alexeyenko A, et al. 2013. The norway spruce genome sequence and conifer genome evolution. *Nature* 497:579–584.

Parra G, Bradnam K, Korf I. 2007. CEGMA: a pipeline to accurately annotate core genes in eukaryotic genomes. *Bioinformatics* 23:1061–1067.

Saitou N, Nei M. 1987. The neighbor-joining method: a new method for reconstructing phylogenetic trees. *Mol. Biol. Evol.* 4:406–425.

Scordis P, Flower DR, Attwood TK. 1999. FingerPRINTScan: intelligent searching of the prints motif database. In: Bioinformatics. Vol. 15. Oxford University Press. p. 799–806.

Shirai K, Matsuda F, Nakabayashi R, Okamoto M, Tanaka M, Fujimoto A, Shimizu M, Shinozaki K, Seki M, Saito K, et al. 2017. A highly specific genome-wide association study integrated with transcriptome data reveals the contribution of copy number variations to specialized metabolites in arabidopsis thaliana accessions. *Mol. Biol. Evol.* 34:3111–3122.

Team RC. 2013. R: a language and environment for statistical computing.

Thompson JD, Gibson TJ, Higgins DG. 2003. Multiple sequence alignment using clustalw and clustalx. *Curr. Protoc. Bioinforma.* 00:2.3.1-2.3.22.

Yang Z. 2007. PAML 4: phylogenetic analysis by maximum likelihood. *Mol. Biol. Evol.*

24:1586–1591.

Yang Z, Nielsen R. 2000. Estimating synonymous and nonsynonymous substitution rates under realistic evolutionary models. *Mol. Biol. Evol.* 17:32–43.
